# Integrative genomic and phenotypic analyses reveal broad-spectrum antifungal activity and biocontrol potential in *Bacillus ayatagriensis*

**DOI:** 10.1007/s10142-026-02063-z

**Published:** 2026-09-29

**Authors:** Vinícius de Souza, Raissa Vitória Viana de Carvalho, Edilania Pereira da Silva, Isaac Farias Cansanção, Edineia Bonin, Leandro Teodoski Spolaor, Adriane Freire Araújo Feitoza, Adailson Feitoza de Jesus Santos

**Affiliations:** 1 Department of Education, State University of Bahia, Paulo Afonso, 48608-230 Brazil; 2 Department of Biological Sciences, State University of Santa Cruz, Ilhéus, 45662-900 Brazil; 3 Campus Paulo Afonso, Federal University of San Francisco Valley, Paulo Afonso, 58605- 780 Brazil; 4 BSS Industry and Trade of Biological Inputs LTDA, Ibiporã, 86200-000 Brazil; 5 Nativus Biotech Consultancy LTDA, Juazeiro, 48907-182 Brazil

**Keywords:** Genome mining, Caatinga biome, Microbial biocontrol, Phytopathogens, Sustainable agriculture

## Abstract

The Caatinga biome represents an important reservoir of microbial biodiversity that remains largely unexplored for the development of agricultural bioinputs. In this study, genomic analyses and phenotypic assays were integrated to investigate the biocontrol potential of two *Bacillus* strains isolated from this environment. Phylogenomic analyses identified the isolates as *Bacillus subtilis* BSS.245 and *Bacillus ayatagriensis* BSS.246. The genomes comprised 4.10 and 4.01 Mb, with GC contents of 43.67% and 46.58%, encoding 4,077 and 3,852 genes, respectively. Genome mining revealed biosynthesis-related gene clusters associated with the production of antimicrobial metabolites, including bacilysin, bacillibactin, fengycin, and surfactin. *B. ayatagriensis* BSS.246 exclusively harbored clusters for macrolactin H and difficidin biosynthesis, compounds widely recognized for their antimicrobial activity. Genes potentially involved in plant growth promotion, stress adaptation, and plant–microbe interactions were also identified. Phenotypic assays demonstrated tolerance to reduced water availability, salinity, and elevated temperature in both strains, as well as exopolysaccharide production. Both strains exhibited broad-spectrum antifungal activity against *Alternaria*, *Colletotrichum*, *Corynespora*, *Fusarium*, *Lasiodiplodia*, *Macrophomina*, *Rhizoctonia*, and *Sclerotinia* species, with mycelial growth inhibition ranging from 58.04% to 100.00%. To our knowledge, this study provides the first experimental evidence of the antifungal potential of *B. ayatagriensis*, expanding the current knowledge of this recently described species. These findings identify both strains as promising agricultural microbial resources and provide a basis for further evaluation of their potential for phytopathogen management and plant-associated applications.

**Supplementary Information:**

The online version contains supplementary material available at https://doi.org/10.1007/s10142-026-02063-z.

## Introduction

Agriculture faces several challenges related to maintaining crop productivity. Pests and diseases are estimated to account for approximately 40% of annual crop losses worldwide, resulting in economic losses of up to US$40 billion (FAO 2021; Ngomle et al. 2025). Among the major phytopathogens, fungi stand out because of their genomic plasticity, broad metabolic versatility, and ability to adapt to diverse environments (Madhushan et al. 2025). These characteristics complicate disease management, as intensive reliance on chemical fungicides promotes the selection of resistant strains while negatively affecting ecosystems and human health (Islam et al. 2024).

In this context, the development of sustainable technologies has become essential for modern agriculture. Microbe-based biocontrol products have emerged as viable alternatives, contributing to increased crop resilience, productivity, and plant health and reduced dependence on chemical fungicides (Lorenzoni et al. 2024; Beyari 2025; Bonser et al. 2025; Tatar and Ali 2025; Sajid and Shabbir 2025). These microorganisms act through multiple mechanisms, including nutrient competition, secondary metabolite production, antagonistic enzyme secretion, siderophore production, volatile organic compounds (VOCs) emission, and plant gene expression modulation (Ons et al. 2020; Legein et al. 2020).

Among the microorganisms most widely employed in microbial biocontrol formulations, species belonging to the genus *Bacillus* have received particular attention (Zhang et al. 2023). The diversity of bioactive metabolites produced by these bacteria, combined with their efficient root colonization and widespread distribution across different ecological niches, has established *Bacillus* species as important tools for sustainable agriculture (Fira et al. 2018; Soni and Keharia 2021; Fatima et al. 2025). Advances in genomic and bioinformatic tools have enabled a more comprehensive exploration of this potential. Computational approaches can accelerate the development of microbial bioinputs by identifying genes and metabolic pathways potentially associated with traits of agronomic interest, thereby supporting the discovery of new and effective biological solutions for agriculture (Rosales-Castillo et al. 2025).

Despite recent advances in microbial genome mining, a significant gap remains between the in silico identification of genes associated with biocontrol traits and the experimental validation of these functions. This limitation reduces the accuracy of selecting promising microbial candidates for bioinput development. Such a gap is particularly evident for microorganisms isolated from extreme and underexplored environments, including the Caatinga biome, which harbors unique microbial diversity with considerable potential for agricultural applications (Dias et al. 2024; Pereira Da Silva et al. 2025; Pereira et al. 2026; De Souza et al. 2026). The environmental conditions of this semiarid ecosystem may favor the selection of microorganisms with specialized metabolic capabilities and enhanced adaptation to environmental stresses. Consequently, these microbial communities represent valuable sources of novel biological resources for sustainable agriculture. In addition, the discovery of new strains with previously unreported functional traits may expand the opportunities for the development of innovative bioinputs.

The advancement of phylogenomic approaches has accelerated the description of new species within the family Bacillaceae, expanding the repertoire of microorganisms with potential agricultural applications. However, the functional attributes of many of these recently described species remain poorly characterized, particularly those related to the biocontrol of phytopathogens. Among them, *Bacillus ayatagriensis* has recently been described and remains largely unexplored, with available studies focusing primarily on plant growth promotion and biomedical applications (Das et al. 2025). To date, no studies have demonstrated the antifungal or biocontrol potential of this species against phytopathogenic fungi.

In the present study, genomic analyses and phenotypic assays were integrated to characterize two *Bacillus* strains isolated from the Caatinga biome. In addition to revealing genetic traits associated with biocontrol and plant–microbe interactions, the results provide the first experimental evidence of the antifungal potential of *B. ayatagriensis*, expanding the current knowledge of this recently described species and highlighting its potential for agricultural applications.

## Materials and methods

### Microorganisms

The bacterial isolates BSS.245 and BSS.246 used in this study were obtained from the microbial collection of the Laboratory of Ecology and Microbial Biotechnology of the Semiarid Region (LEBIMS), State University of Bahia, Paulo Afonso, Brazil. The isolate BSS.245 was obtained as an endophytic strain from bulbils of *Agave sisalana* P., whereas BSS.246 was isolated from the rhizosphere soil of *Tacinga palmadora* (Britton & Rose) N.P. Taylor & Stuppy. Both strains were originally isolated from Serra do Umbuzeiro, Paulo Afonso, Bahia, Brazil (Latitude: −9.58103091088262; Longitude: −38.2047843933105).

### Genomic DNA extraction

The isolates were cultivated in tryptic soy broth (TSB) under constant agitation for 48 h at 28 ± 2 °C. Genomic DNA was extracted using the EasyPure^®^ Bacteria Genomic DNA Kit (TransGen Biotech, Beijing, China) according to the manufacturer’s instructions. DNA quality was assessed by electrophoresis on 1% agarose gels and quantified using a NanoDrop 2000 spectrophotometer (Thermo Scientific, Wilmington, DE, USA).

### Genome sequencing and assembly

The genome of BSS.245 was sequenced using the Illumina NovaSeq 6000 platform with paired-end reads (PE150), whereas the genome of BSS.246 was sequenced using Oxford Nanopore technology on a MinION device equipped with an R10.4.1 flow cell. Sequence quality was evaluated using FastQC v.0.12.1 (Andrews 2010) for BSS.245 and Fastplong v.0.4.1.0 (Chen 2023) for BSS.246. Genome assembly of BSS.245 was performed using Unicycler v.0.5.0 (Wick et al. 2017), while the genome of BSS.246 was assembled using Raven v.1.8.3 (Vaser and Šikić 2021) and subsequently polished with Medaka v.2.1.1. Genome completeness and contamination were assessed using CheckM2 v.1.1.0 (Chklovski et al. 2023). Assembly quality was further evaluated using BUSCO (Benchmarking Universal Single-Copy Orthologs) v.5.8.0 with the bacillales_odb12 dataset (Simão et al. 2015). The genome sequences of BSS.245 and BSS.246 were deposited in GenBank under the accession numbers JCATXW000000000 and JCATXX000000000, respectively.

### Taxonomic and phylogenomic characterization

The 16 S rRNA gene sequences were retrieved from the assembled genomes using Barrnap v.1.2.2 and compared against sequences derived from type material available in the NCBI database using BLASTn. For each isolate, the 100 sequences showing the highest sequence identity were examined to identify the closest species represented among the type material. Reference genomes of the identified species were selected for taxonomic and phylogenomic analysis. Genomic similarity between the isolates and selected type strains was assessed through average nucleotide identity (ANI) analysis using FastANI v.1.3 (Jain et al. 2018). Digital DNA–DNA hybridization (dDDH) analyses and whole-genome phylogenomic reconstruction were performed using the Type Strain Genome Server (TYGS) platform (Meier-Kolthoff and Göker 2019). The resulting phylogenomic trees were visualized and edited using Interactive Tree of Life (iTOL) v.7 (Letunic and Bork 2024). Species delineation was based on accepted thresholds of ANI ≥ 95% and dDDH ≥ 70%. The complete list of reference strains included in the taxonomic and phylogenomic analyses is provided in Supplementary Tables S1 and S2 for BSS.245 and BSS.246, respectively.

### Genome annotation and functional prediction

Genome annotation was performed using Prokka v.1.14.6 (Seemann 2014). The functional categorization of genes into metabolic subsystems was conducted using the RAST (Rapid Annotation using Subsystem Technology) server (Aziz et al. 2008). Annotated genomes were visualized using Proksee v.5.2.1 (Grant et al. 2023) and the BV-BRC/PATRIC platform v.3.58.4 (Olson et al. 2023; Wattam et al. 2017). Biosynthesis-related gene clusters associated with secondary metabolite production were predicted using antiSMASH v.6.1.1 (Blin et al. 2021). Genes associated with plant growth-promoting traits and tolerance to abiotic stresses were predicted using the PGPT-Pred module available in PlaBAse (PLant-associated BActeria web resource). Genes involved in the catabolism of root exudate-derived compounds were identified using rhizoSMASH (rhizosphere-competence-related cataboliSM Analysis SHell) (Li et al. 2025). All analyses were performed using the default parameters of each software tool unless otherwise specified.

### In vitro assessment of plant growth-promoting traits and abiotic stress tolerance

Auxin production was evaluated by the colorimetric method with Salkowski’s reagent at 530 nm, using a calibration curve prepared with IAA (Gordon and Weber 1951). Siderophore production was determined using the chrome azurol S (CAS) colorimetric assay (Schwyn and Neilands 1987). The ability to solubilize phosphate and potassium was determined by halo formation on Pikovskaya and Aleksandrov media, respectively (Pikovskaya 1948; Aleksandrov et al. 1967). EPS production was assessed following the method described by Paulo et al. (2012). Tolerance to water restriction was evaluated by inoculating the strains on TSA medium supplemented with sorbitol at concentrations of 285, 405, 520, and 780 g L⁻¹, corresponding to water activities (Aw) of 0.957, 0.919, 0.897, and 0.807, respectively (Hallsworth et al. 1998). Salt stress tolerance was assessed by inoculating the strains onto TSA supplemented with increasing concentrations of NaCl (0, 2, 4, 6, 8, and 10%, w/v). Thermotolerance was evaluated by incubating the strains on TSA at temperatures ranging from 30 to 50 °C, in 5 °C increments, with 28 °C as the control temperature.

### In vitro antagonism against phytopathogenic fungi

The antagonistic activity of the bacterial strains was evaluated against fungal phytopathogens of agricultural relevance. The assays were conducted using isolates of *Alternaria* sp., *Colletotrichum siamense*, *Corynespora cassiicola*, *Fusarium graminearum*, *Fusarium oxysporum*, *Fusarium solani*, *Lasiodiplodia* sp., *Macrophomina phaseolina*, *Rhizoctonia solani*, and *Sclerotinia sclerotiorum*. Antifungal activity was assessed through direct confrontation assays. For each bacterial strain–fungal pathogen combination, three bacterial suspensions were independently prepared and standardized from the same initial bacterial inoculum to an OD600 of 1.0 (10⁸ CFU mL⁻¹). From each suspension, 100 µL was individually spread onto potato dextrose agar (PDA) plates using the spread-plate method. Subsequently, a 5-mm mycelial plug of each fungal pathogen was placed at the center of the plate. The plates were incubated for seven days in a B.O.D. incubator at 28 ± 2 °C under a 12-h photoperiod. The control treatments consisted of fungal plugs grown on PDA plates without bacterial inoculation. Each bacterial suspension was used to inoculate three replicate plates, with each plate containing an independently prepared fungal mycelial plug, resulting in nine plates per bacterial strain–fungal pathogen. Mycelial growth inhibition (%) was calculated according to the equation: Inhibition (%) = (C − T)/C×100, where C corresponds to the radial growth of the control treatment and T corresponds to fungal radial growth in the presence of the bacterial antagonist. For statistical analysis, the three plates corresponding to each bacterial suspension were averaged, resulting in three suspension-level observations per bacterial strain–fungal pathogen combination (*n* = 3). The experiment was conducted in a single experimental run.

### Statistical analysis

Data were subjected to the Shapiro–Wilk normality test and the homogeneity of variances was evaluated using Levene’s test. In vitro antagonism assays were analyzed separately for each bacterial strain using one-way analysis of variance (ANOVA). For each bacterial strain, the ten fungal pathogens were compared independently based on mycelial growth inhibition (%), using the mean value obtained from the three replicate plates associated with each bacterial suspension as the experimental observation (*n* = 3). The two bacterial strains were analyzed separately. When significant differences were detected (*P* < 0.05), the means were compared using the Scott–Knott test at a 5% level. This procedure was selected to group fungal pathogens according to statistically distinct levels of mycelial growth inhibition, facilitating the characterization of the antagonistic spectrum of each bacterial strain. Statistical analyses were performed using RStudio v.4.3.3 (R Core Team 2024).

## Results

### Genomic analyses identify the isolates as *Bacillus subtilis* BSS.245 and *Bacillus ayatagriensis* BSS.246

Genome assembly resulted in draft genomes of 4.10 Mb and 4.01 Mb for BSS.245 and BSS.246, respectively. Genome assembly quality was supported by the L50 values (2 and 1 for BSS.245 and BSS.246, respectively) and N50 values (1.06 Mb for BSS.245 and 2.09 Mb for BSS.246). The detailed assembly statistics are presented in Table 1.

**Table 1:** Genomic characteristics and assembly statistics of the bacterial isolates

Isolate	Sequencing platform	Genome size (bp)	GC (%)	Contigs	N50	L50	Reads	Coverage
BSS.245	Illumina NovaSeq 6000	4,101,496	43.67	27	1,063,461	2	7,633,164	282X
BSS.246	Nanopore MinION	4,010,786	46.58	4	2,090,753	1	416,359	137X

A genome quality assessment revealed 100% completeness for both genomes, with 0.01% contamination detected in BSS.245 and no contamination detected in BSS.246. BUSCO analysis demonstrated a high recovery rate of conserved orthologs belonging to the order Bacillales. BSS.245 genome contained 427 (99.5%) of the expected orthologs, including 426 (99.3%) single-copy genes and one (0.2%) duplicated ortholog, in addition to one fragmented ortholog (0.2%) and one missing ortholog (0.2%). The genome of BSS.246 contained 425 (99.1%) conserved orthologs and four fragmented orthologs (0.9%), with no missing orthologs detected.

**Fig. 1:**
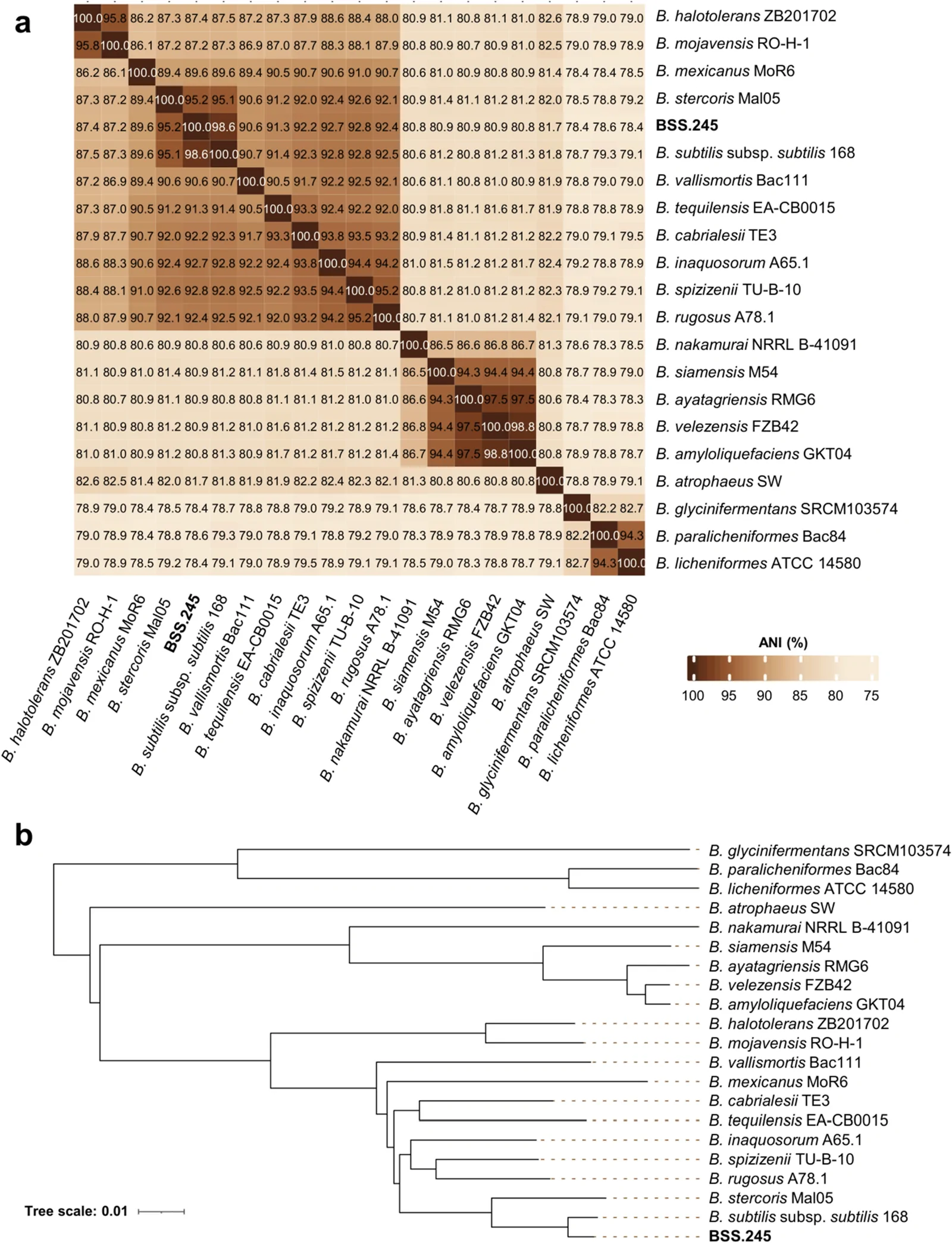
Phylogenomic characterization and genomic similarity of BSS.245. (**a**) Heatmap showing pairwise average nucleotide identity (ANI) values between BSS.245 and closely related *Bacillus* reference genomes. Color intensity represents the degree of genomic similarity, with darker shades indicating higher ANI values. (**b**) Whole-genome phylogenomic tree inferred from genome-wide distances calculated using the Genome BLAST Distance Phylogeny (GBDP) approach. Branch lengths are scaled according to the GBDP *d*5 distance formula. The average branch support was 79.5%. The tree was rooted at the midpoint. Evolutionary distances are represented by the scale bar

BLASTn analysis of the 16 S rRNA gene sequences against type material identified closely related *Bacillus* species for both isolates. The closest matches and their corresponding sequence identities and accession numbers are presented in Supplementary Tables S3 and S4 for BSS.245 and BSS.246, respectively. ANI values indicated that both isolates belonged to the genus *Bacillus* (ANI ≥ 95%). The sequence of the isolate BSS.245 was highly similar to that of *Bacillus subtilis* subsp. *subtilis* 168 (ANI = 98.6%) (Fig. 1a). The taxonomic identity of BSS.245 was further confirmed by dDDH analysis, which yielded values above the species delineation threshold (*d₀* = 91.9, *d₄* = 89.8, *d₆* = 93.9), along with a difference in the G + C content of only 0.16% (Supplementary Table S5). Phylogenomic analysis placed BSS.245 within the *B. subtilis* clade, corroborating its species assignment (Fig. 1b).

**Fig. 2:**
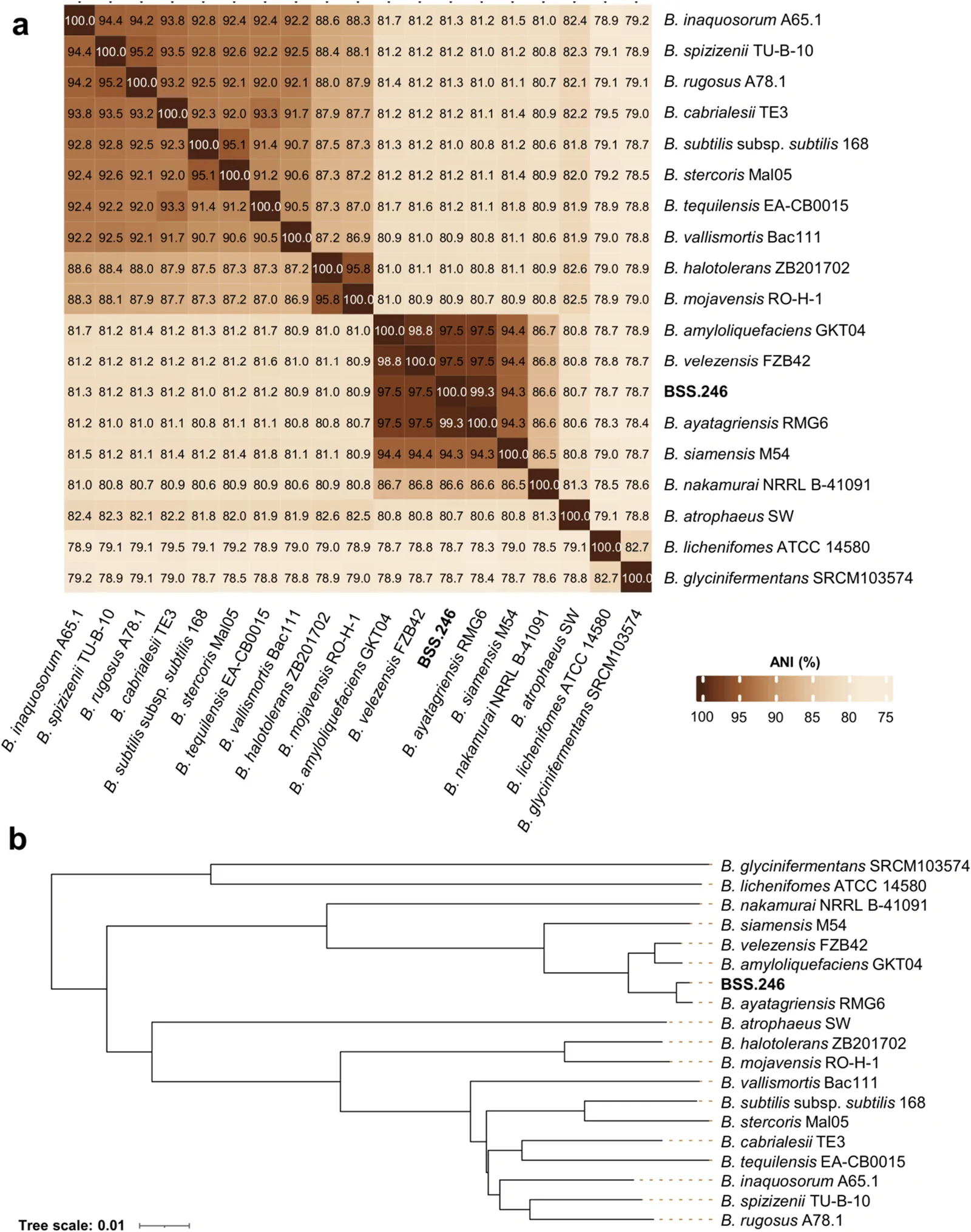
Comparative genomic analysis of the isolate BSS.246 and representative species of the genus *Bacillus*. (**a**) Heatmap showing pairwise average nucleotide identity (ANI) values between BSS.246 and closely related *Bacillus* reference genomes. Color intensity represents the degree of genomic similarity, with darker shades indicating higher ANI values. (**b**) Whole-genome phylogenomic tree inferred from genome-wide distances calculated using the Genome BLAST Distance Phylogeny (GBDP) approach. Branch lengths are scaled according to the GBDP *d*5 distance formula. The average branch support was 84.9%. Evolutionary distances are represented by the scale bar

BSS.246 exhibited high genomic similarity to *Bacillus ayatagriensis* RMG6 (ANI = 99.3%) (Fig. 2a). Lower similarity values were observed with other members of the *Bacillus amyloliquefaciens* species complex, including *Bacillus velezensis* FZB42 (97.5%) and *B. amyloliquefaciens* GKT04 (97.5%). dDDH analysis further confirmed the taxonomic assignment of BSS.246 to *B*. *ayatagriensis*, yielding values of *d₀* = 96.1, *d₄* = 95.5, and *d₆* = 97.5, together with a difference in the G + C content of only 0.05% (Supplementary Table S6). These findings were consistent with the phylogenomic reconstruction, in which BSS.246 clustered closely with *B*. *ayatagriensis*, whereas members of the *B. amyloliquefaciens* complex occupied an adjacent phylogenetic position (Fig. 2b).

### Genomes of *B. subtilis* BSS.245 and *B. ayatagriensis* BSS.246 harbor genes of agronomic interest

**Fig. 3:**
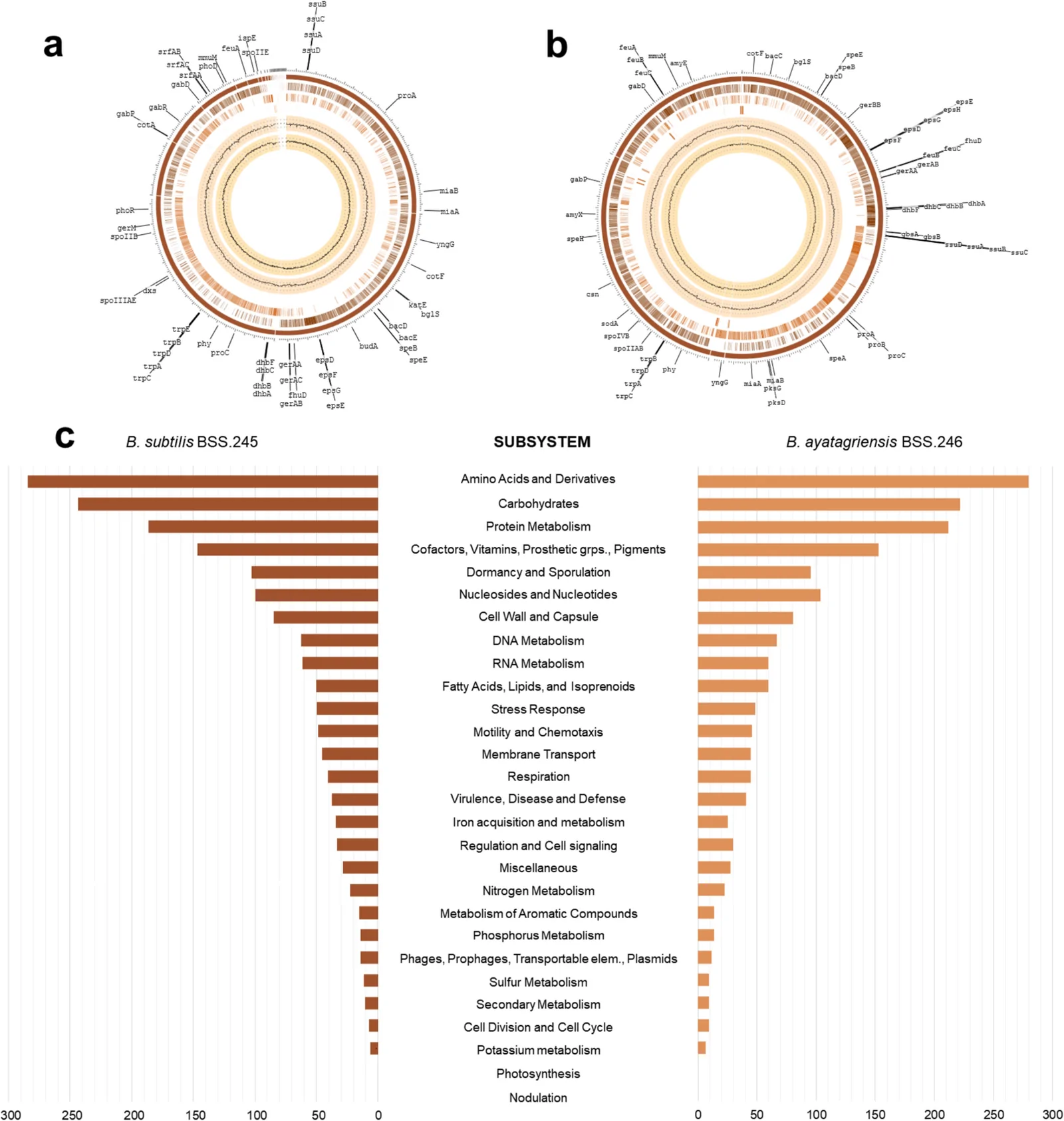
Genomic characterization of the bacterial strains. Circular genome maps of (**a**) *Bacillus subtilis* BSS.245 and (**b**) *Bacillus ayatagriensis* BSS.246 were generated using the Proksee and PATRIC platforms. The genes displayed on the outermost ring correspond to major coding sequences potentially associated with agronomically relevant traits and mechanisms. The concentric rings represent, from the outermost layer to the innermost layer, contigs, CDSs on the forward and reverse strands, RNA genes, GC content, and GC skew. (c) Functional distribution of subsystems annotated by the RAST server for the genomes of BSS.245 and BSS.246. The most represented subsystems included amino acids and derivatives, carbohydrates, protein metabolism, cofactors/vitamins/prosthetic groups/pigments, and dormancy and sporulation, among other functional categories

Genome annotation revealed a total of 4,157 genes in BSS.245, including 4,077 coding DNA sequences (CDSs) and 80 RNA genes, whereas BSS.246 contained 3,950 genes, comprising 3,852 CDSs and 98 RNA genes. Among the predicted CDSs, 1,280 hypothetical proteins were identified in the BSS.245 genome (Fig. 3a), while 1,459 hypothetical proteins were detected in the BSS.246 genome (Fig. 3b).

Functional categorization performed using the RAST server revealed the distribution of genes from both genomes across multiple subsystems associated with metabolic functions and cellular processes (Fig. 3c). Despite differences in genome organization, both strains exhibited a broad repertoire of genes potentially involved in plant–microbe interactions and functions related to biological control (Supplementary Table S7).

### *B. subtilis* BSS.245 and *B. ayatagriensis* BSS.246 harbor genomic features associated with biocontrol potential

**Fig. 4:**
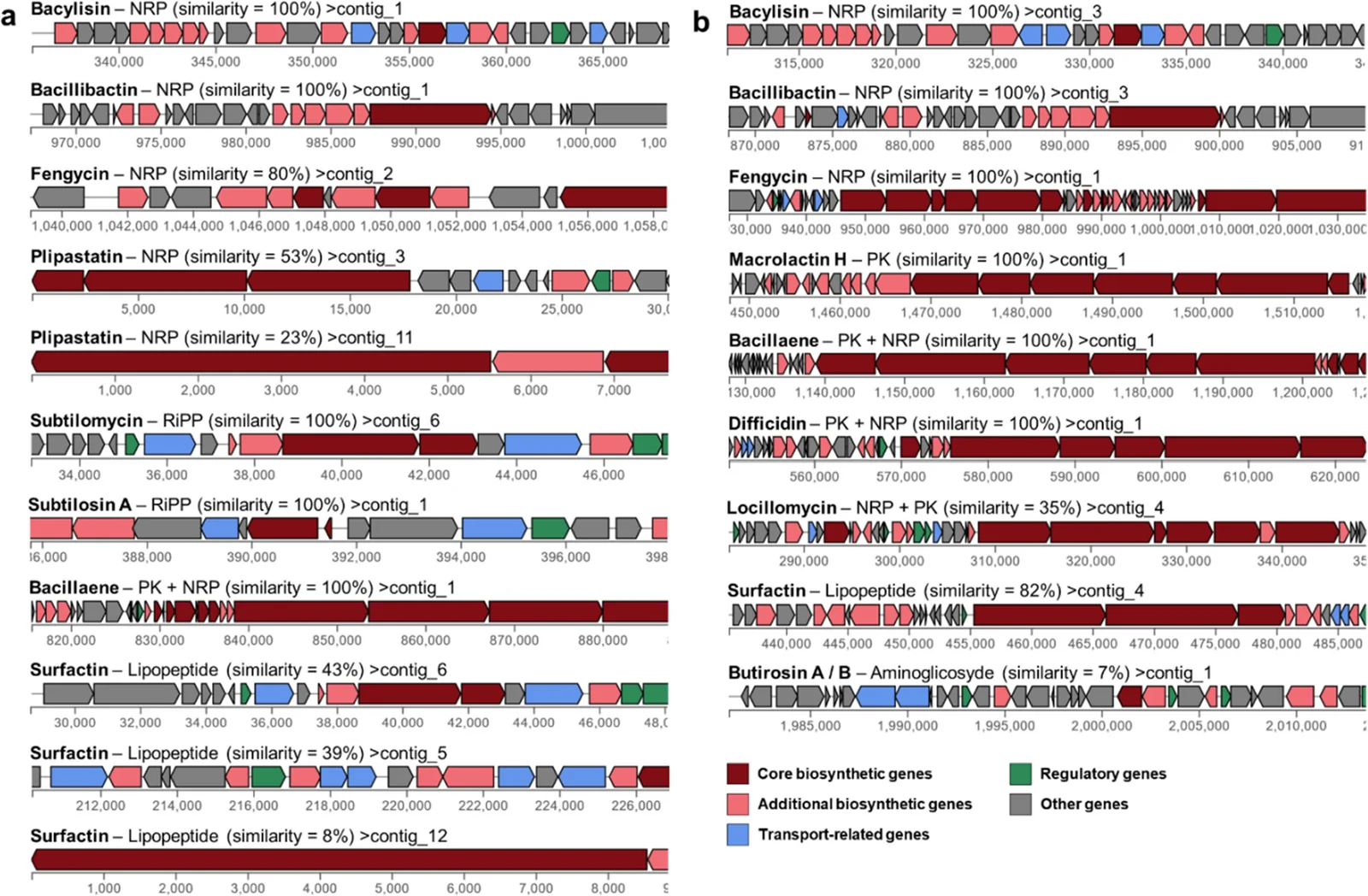
Biosynthetic gene clusters predicted in the genomes of (**a**) *Bacillus subtilis* BSS.245 and (**b**) *Bacillus ayatagriensis* BSS.246. Each cluster is shown according to its genomic location, with the corresponding contig indicated after the similarity value (e.g., > contig_1). The percentages represent the sequence similarity between the predicted biosynthetic gene cluster and the closest reference cluster available in the antiSMASH database. Core biosynthetic genes are shown in dark red, additional biosynthetic genes in pink, transport-related genes in blue, regulatory genes in green, and other genes in gray

Biosynthesis-related gene clusters associated with the production of secondary metabolites were identified in both strains (Fig. 4). The genomes of BSS.245 and BSS.246 exhibited clusters related to the biosynthesis of antimicrobial compounds widely associated with the biological control of phytopathogenic fungi, including bacilysin, bacillibactin, fengycin, and surfactin.

*B. subtilis* BSS.245 contained 11 biosynthesis-related gene clusters potentially involved in the biosynthesis of known secondary metabolites (Fig. 4a). These clusters included ribosomally synthesized and post-translationally modified peptides (RiPPs), including subtilosin A (100%) and subtilomycin (100%); non-ribosomal peptides (NRPs), such as bacillibactin (100%), bacilysin (100%), and fengycin (80%); and bacillaene (100%), a hybrid polyketide–non-ribosomal peptide (PK–NRP) metabolite. Additional clusters with lower similarity were also detected, including those showing similarity to clusters associated with plipastatins (53% and 23%) and surfactins (43%, 39%, and 8%).

*B. ayatagriensis* BSS.246 harbored 10 biosynthesis-related gene clusters that were similar to known secondary metabolites (Fig. 4b). These included the NRPs bacillibactin (100%), bacilysin (100%), and fengycin (100%); the polyketide (PK) macrolactin H (100%); the PK–NRP hybrid metabolites bacillaene (100%) and difficidin (100%); and the lipopeptide surfactin (82%). Notably, gene clusters associated with the biosynthesis of macrolactin H and difficidin were exclusively identified in BSS.246, based on antiSMASH predictions. Additional clusters with lower similarity were also detected, including those associated with the NRP–PK hybrid locillomycin (35%) and butirosin (7%), an aminoglycoside antibiotic.

Genome annotation further revealed the presence of genes encoding hydrolytic enzymes potentially involved in the degradation of fungal cell walls. Both BSS.245 and BSS.246 harbored genes associated with the production of β-glucanases (*bglS*) and chitosanases (*cns*). In contrast, genes encoding chitinases (*chiA*) were identified exclusively in the genome of BSS.246. Genomic features potentially related to indirect biocontrol mechanisms were also identified. Genes involved in siderophore biosynthesis and transport, including *fhuBCDG* and *feuABC*, were detected in both strains. In addition, both genomes contained gene clusters associated with the biosynthesis of volatile organic compounds (VOCs), including acetoin (*alsRST*) and 2,3-butanediol (*budA*), which are metabolites frequently linked to pathogen suppression and beneficial plant–microbe interactions.

### Genes in *B. subtilis* BSS.245 and *B. ayatagriensis* BSS.246 suggest potential mechanisms associated with plant growth promotion and stress response modulation

#### Nutrient acquisition and availability

Genomic analyses revealed the presence of several genes associated with plant growth promotion and potentially involved in plant–microbe interactions. Both strains harbor genes involved in nutrient acquisition and mobilization processes that may contribute to nutrient availability in the plant-associated environment.

Genes encoding phytases (*phy*) and acid (*nagD*) and alkaline phosphatases (*phoA* and *phoD*) were identified in both genomes. These enzymes are commonly associated with phosphorus mobilization in the soil environment. In addition, the genomes contain genes involved in the production of various organic acids, which may contribute to nutrient solubilization and availability. The predicted pathways included the synthesis of acetic (*ackA*), aconitic (*acnA* and *acnB*), butyric (*buk*), carbonic (*cynT*), citric (*citA*), fumaric and succinic (*sdhABC*), gluconic (*gdh*), isocitric (*citB*), ketoglutaric (*icd*), lactic (*ldh*), malic (*mdh*), malonic (*accABCD*), propionic (*prpBDE*), tartaric (*ttuB* and *ttuC*), and valeric (*ilvD*) acids. The genetic potential for formic acid production (*fdhA*) was detected exclusively in *B. ayatagriensis* BSS.246.

Genes related to sulfur metabolism were also identified in both strains, including clusters associated with sulfate reduction (*cysCHIJK*) and the transport and degradation of alkanesulfonates (*ssuABCD*). These metabolic traits may contribute to sulfur cycling in the rhizosphere and potentially increase sulfur availability for plant uptake.

#### Hormonal modulation and plant growth promotion

The genomes of both strains contained genes potentially involved in the synthesis of plant growth-regulating molecules. Genes related to the biosynthesis of tryptophan, a precursor of auxins, were identified, as were genes potentially associated with pathways related to auxins (*iaaT*, *yedL*, and *ysnE*), cytokinins (*miaA* and *miaB*), and spermidines (*speBEG*) biosynthesis and metabolism. Genes related to γ-aminobutyric acid (GABA) metabolism (*gabDPT*) were also identified.

#### Potential traits associated with plant colonization and plant–microbe interactions

Genomic features potentially associated with plant colonization and plant–microbe interactions were also identified. Both strains harbor the *eps* gene cluster, which is associated with exopolysaccharide (EPS) biosynthesis and may contribute to biofilm formation and bacterial establishment on plant surfaces. In addition, genes related to the biosynthesis of volatile organic compounds (VOCs) were identified, including genes involved in isoprene production (*ispDEFH*), which has been reported in the context of plant–microbe interactions and may contribute to signaling processes.

### Genomes of *B. subtilis* BSS.245 and *B. ayatagriensis* BSS.246 harbor genes potentially associated with root exudate utilization

Analysis of predicted gene clusters associated with root exudate catabolism revealed differences between *B. subtilis* BSS.245 and *B. ayatagriensis* BSS.246. Both strains showed high similarity (100%) to clusters related to the utilization of the amino acids glutamine and proline, as well as carbohydrates such as inositol, mannitol, sucrose, trehalose, and xylan. The potential for 3-oxoadipate utilization was also identified in both strains. However, *B. subtilis* BSS.245 exhibited a broader repertoire of genes associated with the catabolism of root exudate-derived compounds, including clusters related to the utilization of fructose, maltose, and GABA, which were not identified in *B. ayatagriensis* BSS.246. These results highlight differences in the repertoire of genes potentially associated with root exudate utilization between the two strains.

#### *B. subtilis* BSS.245 and *B. ayatagriensis* BSS.246 harbor stress response-related genes

The genomic potential for oxidative stress response was indicated by the presence of genes associated with different antioxidant systems. Genes encoding alkyl hydroperoxide reductases (*ahpCF*), catalases (*katAEX*), glutathione peroxidases (*bsaA*), methionine sulfoxide reductases (*msrB*), superoxide dismutases (*sodABC*), and thioredoxin reductases (*trxB*) were identified in both strains. Genes related to the synthesis and metabolism of osmoprotective compounds, including those associated with proline (*proABC*), trehalose (*treCPR*), and glycine betaine (*gbsA* and *gbsB*), were also detected. Additionally, both strains harbored *spo*, *cot*, and *ger* gene clusters associated with endospore formation, coat assembly, and germination. Collectively, the genomes of both strains harbor genes potentially contributing to multiple cellular protection mechanisms against adverse environmental conditions.

#### *B. subtilis* BSS.245 and *B. ayatagriensis* BSS.246 exhibit stress response-related traits

Neither BSS.245 nor BSS.246 showed evidence of plant growth-promoting activity under the evaluated conditions. Auxins and siderophore production were not detected in either strain, and neither strain exhibited phosphate- or potassium-solubilizing activity. Regarding abiotic stress tolerance (Table 2), both strains were able to produce exopolysaccharides (EPS), which is potentially associated with bacterial adaptation to environmental stress. Both strains were able to grow under low water availability, with growth observed up to a water activity (A_w_) of 0.897. *B. subtilis* BSS.245 tolerated NaCl concentrations of up to 6%, whereas *B. aytagriensis* BSS.246 grew at NaCl concentrations of up to 8%. Both strains were able to grow at temperatures of up to 50 °C, indicating tolerance to elevated temperature under the conditions evaluated.

**Table 2:** Abiotic stress tolerance traits of *Bacillus subtilis* BSS.245 and *Bacillus ayatagriensis* BSS.246, including exopolysaccharide (EPS) production, minimum water activity (A_w_) supporting bacterial growth under sorbitol-induced osmotic stress, salinity tolerance under NaCl stress, and maximum temperature supporting bacterial growth. Traits are indicated as positive (+) or negative (−)

Strain	EPS	A_w_				NaCl (%)						Temperature (ºC)					
		0.957	0.919	0.897	0.807	0	2	4	6	8	10	28	30	35	40	45	50
BSS.245	+	+	+	+	-	+	+	+	+	-	-	+	+	+	+	+	+
BSS.246	+	+	+	+	-	+	+	+	+	+	-	+	+	+	+	+	+

#### BSS.245 and BSS.246 inhibit the mycelial growth of agriculturally important fungal pathogens

**Fig. 5:**
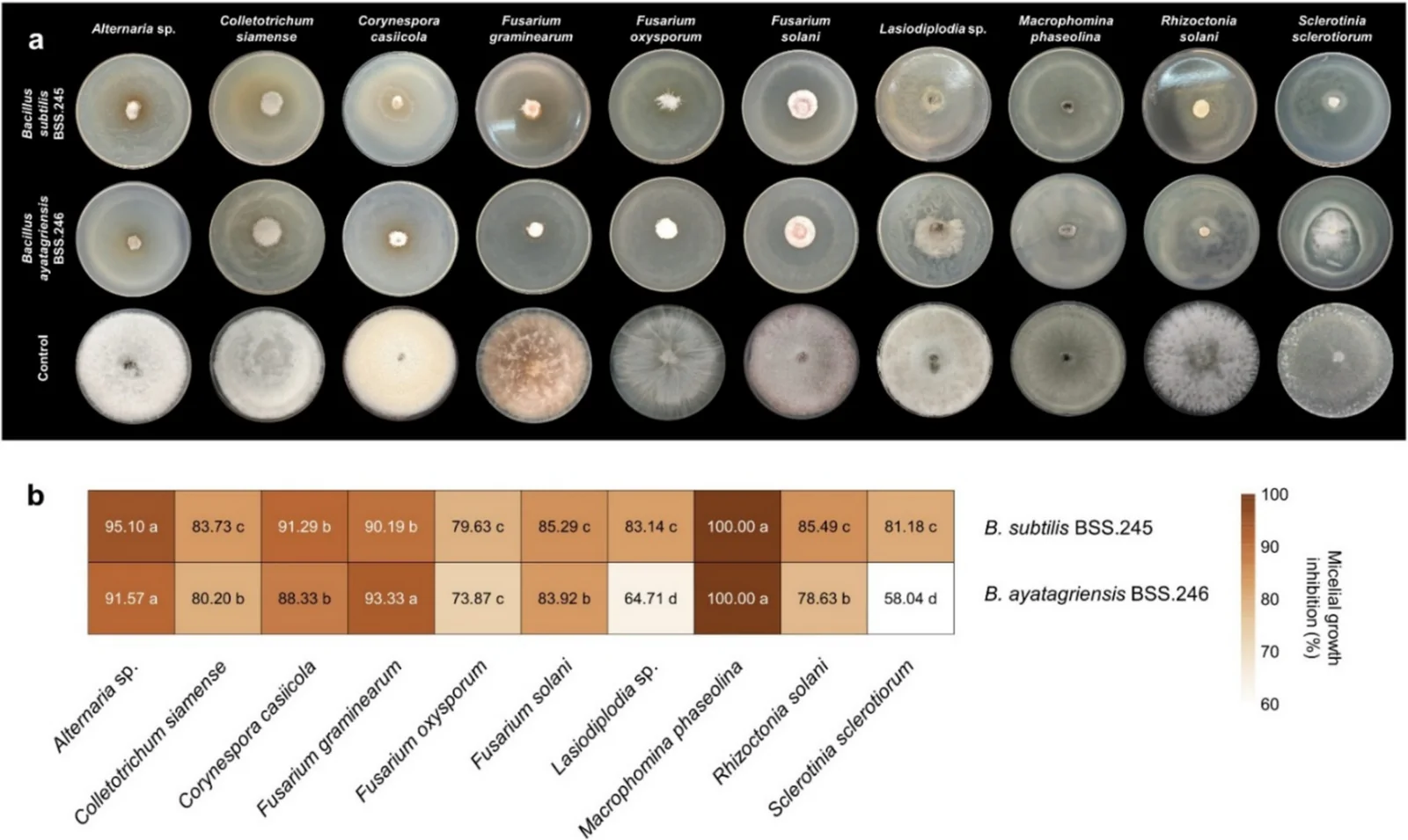
Antifungal activity of *Bacillus subtilis* BSS.245 and *Bacillus ayatagriensis* BSS.246. (**a**) Direct antagonism assay demonstrating the inhibitory effect of the bacterial strains on the mycelial growth of different phytopathogens of agricultural importance. (**b**) Heatmap representing the percentage of mycelial growth inhibition promoted by the strains against the evaluated phytopathogens. The values represent the mean mycelial inhibition (%). Different letters within the same row indicate statistically significant differences among treatments according to the Scott–Knott test (*p* < 0.05)

Both strains exhibited significant antagonistic activity (*p* < 0.001) against all the phytopathogens evaluated (Fig. 5a). Mycelial growth inhibition ranged from 79.62% to 100% for *B. subtilis* BSS.245 and from 58.04% to 100% for *B. ayatagriensis* BSS.246 (Fig. 5b).

Particular attention should be given to the antifungal activity of *B. ayatagriensis* BSS.246, which significantly inhibited the growth of all the evaluated pathogens, with inhibition percentages ranging from 58.04% to 100%. The highest levels of antagonism were observed against *Macrophomina phaseolina* (100 ± 0.00%), *Fusarium graminearum* (93.33 ± 0.52%), *Alternaria* sp. (91.57 ± 0.78%), and *Corynespora cassiicola* (88.33 ± 0.64%). Despite its strong antifungal activity, lower inhibition percentages were recorded against *Lasiodiplodia* sp. (64.71 ± 7.45%) and *Sclerotinia sclerotiorum* (58.04 ± 4.11%).

*B. subtilis* BSS.245 also exhibited strong antagonistic activity, inhibiting the mycelial growth of all the fungal pathogens evaluated. The highest inhibition percentages were observed against *M. phaseolina* (100 ± 0.00%), *Alternaria* sp. (95.10 ± 0.39%), *C. cassiicola* (91.29 ± 1.37%), and *F. graminearum* (90.16 ± 1.04%). Inhibition levels exceeding 70% were also observed against *R. solani*, *F. solani*, *C. siamense*, *Lasiodiplodia* sp., *S. sclerotiorum*, and *F. oxysporum*.

## Discussion

In this study, we integrated genomic analyses and phenotypic assays to characterize two *Bacillus* strains isolated from the Caatinga biome. Our study revealed an extensive repertoire of genes potentially associated with plant growth promotion, adaptation to adverse environmental conditions, and biological control. Furthermore, the results showed that both strains exhibit broad-spectrum antifungal activity against important agricultural pathogens, as well as traits associated with tolerance to abiotic stress. Notably, to the best of our knowledge, this study provides the first experimental evidence of antifungal activity of *B. ayatagriensis* against phytopathogenic fungi. A detailed literature search supporting this finding, including the search strategy and study screening process, is provided in the Supplementary Text S1 and summarized in Supplementary Fig. S1.

Although the genomes were obtained using different sequencing technologies, the analyses indicated that both assemblies were of high quality. Short-read technologies are recognized for their high sequence accuracy, despite their limitations in resolving repetitive regions, which may result in less contiguous assemblies. In contrast, long-read technologies improve assembly continuity and the resolution of repetitive regions, although they may exhibit distinct sequencing error profiles (Pearman et al. 2020; Hotaling et al. 2023). Despite these differences, BUSCO and CheckM analyses demonstrated high genome completeness and low contamination for both strains. These results indicate that both assemblies were suitable for downstream genomic analyses, despite differences in sequencing technologies and assembly approaches. However, we acknowledge that differences between sequencing platforms and assembly pipelines may influence assembly continuity, the resolution of repetitive regions, and contig organization, although apparent inconsistencies were not observed in the resulting predictions or species identification.

*Bacillus ayatagriensis* was recently described as a plant growth-promoting bacterium associated with the rhizosphere of mulberry (*Morus* sp.) (Das et al. 2025). Although the original study reported traits related to plant growth promotion, information regarding its potential for fungal biocontrol was lacking. In the present study, strain BSS.246 exhibited antagonistic activity against all the phytopathogens evaluated and harbored a broad repertoire of genes associated with biocontrol and plant–microbe interactions. These findings substantially expand the current knowledge of this species and support its potential for further evaluation as a multifunctional bioinput candidate. In contrast, strains belonging to *Bacillus subtilis* are widely recognized for their ability to promote plant growth and suppress phytopathogens, with extensive evidence supporting their agricultural applications (Ayaz et al. 2024; Wang et al. 2023; Younis et al. 2024).

The identification of biosynthesis-related gene clusters with similarity to clusters associated with bacilysin, bacillibactin, fengycin, surfactin, and subtilosin biosynthesis suggests the biosynthetic potential of both strains, as these metabolites are frequently associated with phytopathogen suppression and the ecological competitiveness of *Bacillus* species (Salazar et al. 2023; Shahid et al. 2021). Furthermore, the presence of macrolactin H and difficidin biosynthetic clusters in *B. ayatagriensis* BSS.246 suggests a broader biosynthetic repertoire for this strain. Their genomic occurrence supports the presence of biosynthetic potential that may be relevant to the antagonistic phenotype observed in the assays (Sun et al. 2025). However, their production and contribution to the antifungal phenotype remain unconfirmed.

The genomic and phenotypic analyses provided complementary evidence of the biocontrol potential of both strains, with the antagonism assays demonstrating activity against a broad range of phytopathogens. The observed spectrum included fungi associated with root diseases, such as *Fusarium* spp., *M. phaseolina*, *R. solani*, and *S. sclerotiorum*, as well as pathogens associated with aerial plant infections, including *Alternaria* sp., *C. siamense*, *C. cassiicola*, *F. graminearum*, and *Lasiodiplodia* sp. (Singh et al. 2025; Todorović et al. 2023; Sarenqimuge et al. 2025; Vilela et al. 2024; Yang et al. 2025). Collectively, these findings indicate broad antagonistic activity in vitro.

The genes identified in the BSS.245 and BSS.246 genomes suggest genetic potential for traits associated with plant growth promotion. However, despite the genomic predictions, these traits were not detected in vitro. This discrepancy highlights that the presence of genes associated with a given function does not necessarily result in phenotypic expression under specific experimental conditions. Therefore, these genomic features should be interpreted as functional potential rather than demonstrated plant growth-promoting activity, and experimental validation under relevant conditions is required to determine their biological significance (Wei et al. 2025).

The differences observed in the repertoire of genes associated with root exudate utilization suggest potential differences in the metabolic capacities of the two strains. *B. subtilis* BSS.245 exhibited a broader repertoire of genes related to the assimilation of root-derived compounds, potentially indicating a greater genomic capacity to utilize diverse compounds available in the rhizosphere (Compant et al. 2021). The more restricted repertoire observed in BSS.246 may indicate a potentially narrower genomic capacity for the utilization of root-derived compounds, which could be related to its native ecological niche (Thamizharasan et al. 2025). However, these interpretations represent genomic potential rather than demonstrated metabolic functions.

The repertoire of genes associated with osmoregulation and oxidative stress responses identified suggests potential genetic mechanisms contributing to tolerance to adverse environmental conditions. Genes potentially involved in osmotic adjustment and antioxidant responses were detected, consistent with mechanisms involved in maintaining cellular homeostasis under water deficit, salinity, high temperatures, and oxidative stress (Bashir et al. 2014). These genomic features were supported by the phenotypic assays, which confirmed tolerance to reduced water availability, salinity, and elevated temperatures, indicating that both strains tolerated multiple abiotic stresses under the conditions evaluated. These traits may be particularly relevant in the Caatinga biome, characterized by high temperatures, intense solar radiation, and irregular rainfall patterns (Lacerda-Júnior et al. 2019).

Although this study demonstrated promising genomic and phenotypic traits, further studies are required to validate the functional potential of these strains. In particular, metabolomic and targeted biochemical approaches could determine whether the predicted biosynthetic pathways are expressed and contribute to the antifungal phenotype observed in vitro. Greenhouse and field experiments will also be necessary to determine whether the observed antagonistic activity and abiotic stress tolerance translate into effective biocontrol under agricultural conditions. Such studies will help establish the functional relevance and practical potential of BSS.245 and BSS.246 as microbial resources for agriculture.

In conclusion, the integration of genomic and phenotypic analyses revealed that *B. subtilis* BSS.245 and *B. ayatagriensis* BSS.246 harbor genomic features potentially associated with biocontrol, plant growth promotion, and tolerance to adverse environmental conditions. Both strains exhibited broad-spectrum antagonistic activity against important phytopathogens and tolerance to abiotic stresses. Notably, this study provides the first experimental evidence of antifungal activity of *B. ayatagriensis* against phytopathogenic fungi and expands the current knowledge of its functional potential. Overall, the genomic and phenotypic profiles identify BSS.245 and BSS.246 as strains of interest for further evaluation as agricultural microbial resources. However, validation under greenhouse and field conditions, together with additional studies, will be necessary to determine their effectiveness and suitability for development as multifunctional bioinputs.

## Supplementary Information

Below is the link to the electronic supplementary material.

Supplementary Material 1 (PDF 1.21 MB)

## Data Availability

The genome sequences of BSS.245 and BSS.246 were deposited in GenBank under the accession numbers JCATXW000000000 and JCATXX000000000, respectively.
