# Supplementary material for: Integrative genomic and phenotypic analyses reveal broad-spectrum antifungal activity and biocontrol potential in Bacillus ayatagriensis
Source: Funct Integr Genomics. 2026 Sep 29;26(1):275. doi: 10.1007/s10142-026-02063-z (PMC13623793; doi:10.1007/s10142-026-02063-z)

Integrative genomic and phenotypic analyses reveal broad-spectrum antifungal activity and biocontrol potential in *Bacillus ayatagriensis*

Vinícius de Souza[1], Raissa Vitória Viana de Carvalho[1], Edilania Pereira da Silva[2], Isaac Farias Cansanção[3], Edineia Bonin[4], Leandro Teodoski Spolaor[4], Adriane Freire Araújo Feitoza[5], Adailson Feitoza de Jesus Santos[1*]

[1] Department of Education, State University of Bahia, Paulo Afonso, 48608-230, Brazil
[2] Department of Biological Sciences, State University of Santa Cruz, Ilhéus, 45662-900, Brazil

[3] Campus Paulo Afonso, Federal University of San Francisco Valley, Paulo Afonso, 58605-780, Brazil

[4] BSS Industry and Trade of Biological Inputs LTDA, Ibiporã, 86200-000, Brazil

[5] Nativus Biotech Consultancy LTDA, Juazeiro, 48907-182, Brazil

*Corresponding author: afsantos@uneb.br

SUPPLEMENTARY MATERIAL

Supplementary Table S1. Genomic and taxonomic characteristics of the reference *Bacillus* strains included in the comparative analysis of BSS.245. The table provides assembly identifiers, GenBank and RefSeq accession numbers, species and strain designations, genome size (bp), number of contigs, GC content, and genome completeness and contamination estimates obtained using CheckM. The reference genomes were selected based on their taxonomic relatedness to BSS.245 and were used for comparative genomic analyses, including ANI, dDDH, and phylogenomic reconstruction.

**Assembly**	**GenBank**	**RefSeq**	**Species**	**Strain**	**bp**	**Contigs**	**GC (%)**	**CheckM completeness**	**CheckM contamination**
ASM1939692v1	GCA_019396925.1	GCF_019396925.1	*B. amyloliquefaciens*	GKT04	4149690	2	46.5	97.82	0.86
ASM3951917v1	GCA_039519175.1	GCF_039519175.1	*B. atrophaeus*	SW	4292910	2	43	99.12	1.18
ASM4019437v1	GCA_040194375.1	GCF_040194375.1	*B. ayatagriensis*	RMG6	3821630	37	46.5	99.41	0.0
ASM412431v2	GCA_004124315.2	GCF_004124315.2	*B. cabrialesii*	TE3	4125766	1	44	98.79	0.0
ASM410361v1	GCA_004103615.1	GCF_004103615.1	*B. glycinifermentans*	SRCM103574	4810226	2	46	97.6	0.0
ASM400643v1	GCA_004006435.1	GCF_004006435.1	*B. halotolerans*	ZB201702	4154245	1	44	98.82	0.59
ASM2323832v1	GCA_023238325.1	GCF_023238325.1	*B. inaquosorum*	A65.1	4242130	1	44	98.82	0.0
ASM3447892v1	GCA_034478925.1	GCF_034478925.1	*B. licheniformis*	ATCC 14580	4227253	1	46	98.5	0.83
ASM5044561v1	GCA_050445615.1	GCF_050445615.1	*B. mexicanus*	MoR6	4299957	27	41	99.41	6.83
ASM5663218v1	GCA_056632185.1	GCF_056632185.1	*B. mojavensis*	RO-H-1	3976981	1	44	98.53	0.0
ASM158432v1	GCA_001584325.1	GCF_001584325.1	*B. nakamurai*	NRRL B-41091	3689277	45	45.5	99.26	0.0
ASM299392v1	GCA_002993925.1	GCF_002993925.1	*B. paralicheniformis*	Bac84	4376831	1	46	98.78	0.0
ASM2323824v1	GCA_023238245.1	GCF_023238245.1	*B. rugosus*	A78.1	4146938	1	44	98.82	0.0
ASM5162807v1	GCA_051628075.1	GCF_051628075.1	*B. siamensis*	M54	3897476	1	46.5	99.41	0.0
ASM22746v1	GCA_000227465.1	GCF_000227465.1	*B. spizizenii*	TU-B-10	4207222	1	44	98.29	0.29
ASM2977298v1	GCA_029772985.1	GCF_029772985.1	*B. stercoris*	Mal05	4282228	1	43.5	99.2	0.51
ASM904v1	GCA_000009045.1	GCF_000009045.1	*B. subtilis* subsp. *Subtilis*	168	4215606	1	43.5	98.62	1.32
ASM1222588v1	GCA_012225885.1	GCF_012225885.1	*B. tequilensis*	EA-CB0015	4012371	1	43.5	98.82	0.0
ASM366788v1	GCA_003667885.1	GCF_003667885.1	*B. vallismortis*	Bac111	3958372	1	44	99.35	0.0
ASM1578v2	GCA_000015785.2	GCF_000015785.2	*B. velezensis*	FZB42	3918596	1	46.5	99.41	0.0

Supplementary Table S2. Genomic and taxonomic characteristics of the reference *Bacillus* strains included in the comparative analysis of BSS.246. The table provides assembly identifiers, GenBank and RefSeq accession numbers, species and strain designations, genome size (bp), number of contigs, GC content, and genome completeness and contamination estimates obtained using CheckM. The reference genomes were selected based on their taxonomic relatedness to BSS.246 and were used for comparative genomic analyses, including ANI, dDDH, and phylogenomic reconstruction.

Assembly	GenBank	RefSeq	Species	Strain	bp	Contigs	GC (%)	CheckM completeness	CheckM contamination
ASM904v1	GCA_000009045.1	GCF_000009045.1	*B. subtilis* subsp. *subtilis*	168	4215606	1	43.5	98.62	1.32
ASM1578v2	GCA_000015785.2	GCF_000015785.2	*B. velezensis*	FZB42	3918596	1	46.5	99.41	0.0
ASM3447892v1	GCA_034478925.1	GCF_034478925.1	*B. licheniformis*	ATCC 14580	4227253	1	46	98.5	0.83
ASM22746v1	GCA_000227465.1	GCF_000227465.1	*B. spizizenii*	TU-B-10	4207222	1	44	98.29	0.29
ASM5663218v1	GCA_056632185.1	GCF_056632185.1	*B. mojavensis*	RO-H-1	3976981	1	44	98.53	0.0
ASM412431v2	GCA_004124315.2	GCF_004124315.2	*B. cabrialesii*	TE3	4125766	1	44	98.79	0.0
ASM1939692v1	GCA_019396925.1	GCF_019396925.1	*B. amyloliquefaciens*	GKT04	4149690	2	46.5	97.82	0.86
ASM400643v1	GCA_004006435.1	GCF_004006435.1	*B. halotolerans*	ZB201702	4154245	1	44	98.82	0.59
ASM3951917v1	GCA_039519175.1	GCF_039519175.1	*B. atrophaeus*	SW	4292910	2	43	99.12	1.18
ASM5162807v1	GCA_051628075.1	GCF_051628075.1	*B. siamensis*	M54	3897476	1	46.5	99.41	0.0
ASM2323832v1	GCA_023238325.1	GCF_023238325.1	*B. inaquosorum*	A65.1	4242130	1	44	98.82	0.0
ASM2977298v1	GCA_029772985.1	GCF_029772985.1	*B. stercoris*	Mal05	4282228	1	43.5	99.2	0.51
ASM366788v1	GCA_003667885.1	GCF_003667885.1	*B. vallismortis*	Bac111	3958372	1	44	99.35	0.0
ASM410361v1	GCA_004103615.1	GCF_004103615.1	*B. glycinifermentans*	SRCM103574	4810226	2	46	97.6	0.0
ASM1222588v1	GCA_012225885.1	GCF_012225885.1	*B. tequilensis*	EA-CB0015	4012371	1	43.5	98.82	0.0
ASM2323824v1	GCA_023238245.1	GCF_023238245.1	*B. rugosus*	A78.1	4146938	1	44	98.82	0.0
ASM4019437v1	GCA_040194375.1	GCF_040194375.1	*B. ayatagriensis*	RMG6	3821630	37	46.5	99.41	0.0
ASM158432v1	GCA_001584325.1	GCF_001584325.1	*B. nakamurai*	NRRL B-41091	3689277	45	45.5	99.26	0.0

Supplementary Table S3. BLASTn results of the 16S rRNA gene sequence of BSS.245 against sequences from type material available in the NCBI database. The table presents the closest matches, including the scientific name, maximum and total alignment scores, query coverage, E-value, percentage identity, sequence length, and accession number. These results were used as the initial molecular evidence for the taxonomic assignment of BSS.245.

Description	Scientific Name	Max Score	Total Score	Query Cover	E value	Per. Ident	Acc. Len	Accession
B. subtilis subsp. *subtilis* str. 168 genome assembly, chromosome: 1	*B. subtilis* subsp. *subtilis* str. 168	2337	23282	100%	0.0	100.00%	4215606	AL009126.3
B. subtilis subsp. *subtilis* strain KCTC 3135, complete genome	*B. subtilis* subsp. *subtilis*	2337	23354	100%	0.0	100.00%	4211343	CP015375.1
B. subtilis strain NCIB_3610 chromosome, complete genome	*B. subtilis*	2337	23336	100%	0.0	100.00%	4215634	CP130597.1
B. subtilis subsp. *subtilis* str. 168 chromosome, complete genome	*B. subtilis* subsp. *subtilis* str. 168	2337	23343	100%	0.0	100.00%	4227167	CP051860.2
B. cabrialesii strain TE3 16S ribosomal RNA, complete sequence	*B. cabrialesii*	2337	2337	100%	0.0	100.00%	1550	NR_180419.1
B. subtilis subsp. *subtilis* strain DSM 10 chromosome, complete genome	*B. subtilis* subsp. *subtilis*	2337	23336	100%	0.0	100.00%	4215636	CP060710.1
B. subtilis strain NCIB 3610 chromosome, complete genome	*B. subtilis*	2337	23336	100%	0.0	100.00%	4215607	CP020102.1
B. subtilis strain DSM 10 chromosome, complete genome	*B. subtilis*	2337	23343	100%	0.0	100.00%	4215627	CP120681.2
Chain a, 16S rRNA	*B. subtilis* subsp. *subtilis* str. 168	2337	2337	100%	0.0	100.00%	1554	8BUU_a
B. subtilis subsp. *subtilis* str. 168, partial genome	*B. subtilis* subsp. *subtilis* str. 168	2337	22588	100%	0.0	100.00%	4215398	CP019662.1
B. subtilis subsp. *subtilis* str. 168, partial genome	*B. subtilis* subsp. *subtilis* str. 168	2337	23040	100%	0.0	100.00%	4215385	CP019663.1
B. subtilis subsp. *subtilis* str. 168 chromosome, complete genome	*B. subtilis* subsp. *subtilis* str. 168	2337	23343	100%	0.0	100.00%	4398844	CP052842.1
B. subtilis subsp. *spizizenii* TU-B-10, complete genome	*B. spizizenii* TU-B-10	2337	23349	100%	0.0	100.00%	4207222	CP002905.1
B. cabrialesii strain TE3 chromosome, complete genome	*B. cabrialesii*	2337	23371	100%	0.0	100.00%	4125766	CP096889.1
B. subtilis subsp. *subtilis* str. 168 chromosome, complete genome	*B. subtilis* subsp. *subtilis* str. 168	2337	23343	100%	0.0	100.00%	4215167	CP103783.1
B.. sp. FSQ1 16S ribosomal RNA gene, partial sequence	*B. mexicanus*	2337	2337	100%	0.0	100.00%	1349	ON532842.1
B. subtilis strain NCIB 3610 chromosome, complete genome	*B. subtilis*	2337	23371	100%	0.0	100.00%	4210909	CP094361.1
B. subtilis subsp. *subtilis* str. 168 chromosome, complete genome	*B. subtilis* subsp. *subtilis* str. 168	2337	23371	100%	0.0	100.00%	4217417	CP136402.1
B. cabrialesii strain TE3 16S ribosomal RNA gene, partial sequence	*B. cabrialesii*	2337	2337	100%	0.0	100.00%	1550	MK462260.1
B. rugosus strain SPB7 16S ribosomal RNA gene, partial sequence	*B. rugosus*	2337	2337	100%	0.0	100.00%	1548	MT554518.1
B. subtilis subsp. *subtilis* str. 168 chromosome, complete genome	*B. subtilis* subsp. *subtilis* str. 168	2337	23343	100%	0.0	100.00%	4316079	CP053102.1
B. subtilis gene, 180 kilobase region of replication origin	*B. subtilis* subsp. *subtilis* str. 168	2337	9337	100%	0.0	100.00%	180136	D26185.1
B. rugosus strain SPB7 16S ribosomal RNA, partial sequence	*B. rugosus*	2337	2337	100%	0.0	100.00%	1548	NR_181236.1

B. subtilis subsp. *subtilis* 6051-HGW, complete genome	*B. subtilis* subsp. *subtilis* 6051-HGW	2337	23282	100%	0.0	100.00%	4215610	CP003329.1
B. subtilis subsp. *subtilis* strain 168 chromosome, complete genome	*B. subtilis* subsp. *subtilis*	2337	23343	100%	0.0	100.00%	4215502	CP171645.1
B. subtilis subsp. *subtilis* NCIB 3610 = ATCC 6051 = DSM 10 strain NCIB 3610 chromosome, complete genome	*B. subtilis* subsp. *subtilis* NCIB 3610 = ATCC 6051 = DSM 10	2337	23371	100%	0.0	100.00%	4211262	CP034484.1
B. subtilis subsp. *subtilis* str. 168, complete genome	*B. subtilis* subsp. *subtilis* str. 168	2337	23336	100%	0.0	100.00%	4215619	CP010052.1
B. subtilis subsp. *subtilis* NBRC 13719 DNA, complete genome	*B. subtilis* subsp. *subtilis*	2337	23349	100%	0.0	100.00%	4211090	AP019714.1
B. inaquosorum strain KCTC 13429 chromosome, complete genome	*B. inaquosorum*	2337	20986	100%	0.0	100.00%	4350498	CP029465.1
B. subtilis strain DSM 10 chromosome, complete genome	*B. subtilis*	2337	23371	100%	0.0	100.00%	4211264	CP149576.1
B. subtilis subsp. *subtilis* str. 168 chromosome, complete genome	*B. subtilis* subsp. *subtilis* str. 168	2337	23365	100%	0.0	100.00%	4217427	CP147877.1
B. stercoris strain JCM 30051 16S ribosomal RNA gene, partial sequence	*B. stercoris*	2335	2335	100%	0.0	100.00%	1548	PP239385.1
B. inaquosorum strain BGSC 3A28 16S ribosomal RNA, partial sequence	*B. inaquosorum*	2335	2335	100%	0.0	100.00%	1538	NR_104873.1
Chain a, 16S ribosomal RNA	*B. subtilis* subsp. *subtilis* str. 168	2331	2331	100%	0.0	99.92%	1554	6HA1_a
Chain AA, 16S ribosomal RNA	*B. subtilis* subsp. *subtilis* str. 168	2331	2331	100%	0.0	99.92%	1555	3J9W_AA
B. subtilis DNA for phoB-rrnE-groESL region, complete cds	*B. subtilis*	2331	2331	100%	0.0	99.92%	36308	D88802.1
B. subtilis strain IAM 12118 16S ribosomal RNA, complete sequence	*B. subtilis*	2331	2331	100%	0.0	99.92%	1550	NR_112116.2
B. subtilis strain IAM 12118 16S ribosomal RNA gene, partial sequence	*B. subtilis*	2331	2331	100%	0.0	99.92%	1550	MZ315033.1
B. subtilis subsp. *subtilis* strain 168 16S ribosomal RNA, complete sequence	*B. subtilis* subsp. *subtilis*	2331	2331	100%	0.0	99.92%	1550	NR_102783.2
B. spizizenii ATCC 6633 = JCM 2499 strain ATCC 6633 chromosome, complete genome	*B. spizizenii* ATCC 6633 = JCM 2499	2331	23315	100%	0.0	99.92%	4045538	CP034943.1
B. subtilis gene for 16S ribosomal RNA, partial sequence	*B. subtilis*	2331	2331	100%	0.0	99.92%	1553	AB042061.1
Chain A, 16S ribosomal RNA	*B. subtilis* subsp. *subtilis* str. 168	2329	2329	100%	0.0	99.92%	1544	5NJT_A
B. subtilis subsp. *subtilis* strain 168 16S ribosomal RNA gene, partial sequence	*B. subtilis* subsp. *subtilis*	2329	2329	100%	0.0	99.92%	1550	PQ164258.1
B. vallismortis strain DSM 11031 chromosome, complete genome	*B. vallismortis*	2326	23199	100%	0.0	99.84%	4286362	CP026362.1
B. subtilis genome, trnI-feuABC region	*B. subtilis* subsp. *subtilis* str. 168	2326	4631	100%	0.0	99.84%	15467	D84213.1
B. vallismortis strain DV1-F-3 chromosome, complete genome	*B. vallismortis*	2326	23199	100%	0.0	99.84%	4234100	CP159908.1
Chain a, 16S ribosomal RNA	*B. subtilis* subsp. *subtilis* str. 168	2324	2324	100%	0.0	99.84%	1545	7QV2_a
B. subtilis 25 kb genomic DNA segment (from sspE to katA)	*B. subtilis* subsp. *subtilis* str. 168	2324	2324	100%	0.0	99.84%	24524	Z82044.1
B. mojavensis RO-H-1 = KCTC 3706 strain RO-H-1 chromosome, complete genome	*B. mojavensis* RO-H-1 = KCTC 3706	2320	20829	100%	0.0	99.76%	3976981	CP196936.1

B. halotolerans strain DSM 8802 16S ribosomal RNA, partial sequence	*B. halotolerans*	2320	2320	100%	0.0	99.76%	1545	NR_115063.1
B. subtilis genes for ribosomal proteins L13 and S9, putative cell wall hydrolase CwlD, gerD protein, 16S ribosomal RNA and 23S ribosomal RNA	*B. subtilis* subsp. *subtilis* str. 168	2318	2318	100%	0.0	99.76%	13119	D64126.1
B. amyloliquefaciens DSM7 complete genome	*B. amyloliquefaciens* DSM 7 = ATCC 23350	2309	23088	100%	0.0	99.60%	3980199	FN597644.1
Chain a, 16S rRNA	*B. subtilis* subsp. *subtilis* str. 168	2309	2309	99%	0.0	99.92%	1533	6HTQ_a
B. ayatagriensis strain RMG6 16S ribosomal RNA gene, partial sequence	*B. ayatagriensis*	2309	2309	100%	0.0	99.60%	1545	PP494329.1
B. subtilis strain DSM 10 16S ribosomal RNA gene, partial sequence	*B. subtilis*	2307	2307	99%	0.0	100.00%	1513	MW049219.1
B. subtilis strain DSM 10 16S ribosomal RNA gene, partial sequence	*B. subtilis*	2307	2307	99%	0.0	100.00%	1510	MN077147.1
B. subtilis strain DSM 10 16S ribosomal RNA, partial sequence	*B. subtilis*	2307	2307	99%	0.0	100.00%	1517	NR_027552.1
B. velezensis strain BCRC 17467 16S ribosomal RNA gene, partial sequence	*B. velezensis*	2305	2305	100%	0.0	99.68%	1535	EF433407.1
B. vallismortis strain DSM 11031 16S ribosomal RNA, partial sequence	*B. vallismortis*	2305	2305	99%	0.0	99.76%	1530	NR_024696.1
B. mojavensis strain IFO 15718 16S ribosomal RNA, partial sequence	*B. mojavensis*	2300	2300	99%	0.0	99.68%	1526	NR_024693.1
B. subtilis gene for 16S rRNA, partial sequence, strain: ATCC 6633	*B. spizizenii* ATCC 6633 = JCM 2499	2298	2298	99%	0.0	99.92%	1507	AB018486.1
B. subtilis partial 16S rRNA gene, type strain DSM 10T	*B. subtilis*	2298	2298	98%	0.0	100.00%	1502	LN681568.1
B. inaquosorum strain BGSC 3A28 16S ribosomal RNA gene, partial sequence	*B. inaquosorum*	2274	2274	98%	0.0	99.92%	1501	PX218924.1
B. subtilis subsp. *subtilis* str. 168 16S ribosomal RNA gene, partial sequence	*B. subtilis* subsp. *subtilis* str. 168	2274	2274	98%	0.0	99.92%	1518	PV257656.1
B. stercoris strain JCM 30051 16S ribosomal RNA, partial sequence	*B. stercoris*	2272	2272	97%	0.0	100.00%	1508	NR_180796.1
B. tequilensis strain KCTC 13622(T) 16S ribosomal RNA gene, partial sequence	*B. tequilensis*	2272	2272	98%	0.0	99.84%	1518	MW009674.1
B. subtilis subsp. *stercoris* strain JCM 30051 16S ribosomal RNA gene, partial sequence	*B. stercoris*	2272	2272	97%	0.0	100.00%	1508	MN536904.1
B. subtilis subsp. *spizizenii* strain NRRL B-23049 16S ribosomal RNA gene, partial sequence	*B. spizizenii*	2272	2272	97%	0.0	100.00%	1508	MN536905.1
B. subtilis strain IAM 12118 16S ribosomal RNA gene, partial sequence	*B. subtilis*	2268	2268	98%	0.0	99.84%	1517	PV616805.1
B. subtilis strain IAM 12118 16S ribosomal RNA gene, partial sequence	*B. subtilis*	2268	2268	98%	0.0	99.84%	1521	PV523860.1
B. atrophaeus strain JCM 9070 16S ribosomal RNA, partial sequence	*B. atrophaeus*	2266	2266	99%	0.0	99.20%	1515	NR_024689.1
B. subtilis strain IAM 12118 16S ribosomal RNA gene, partial sequence	*B. subtilis*	2265	2265	97%	0.0	100.00%	1519	MK267098.1
B. nematocida strain B-16 16S ribosomal RNA, partial sequence	*B. nematocida*	2263	2263	98%	0.0	99.52%	1511	NR_115325.1

B. rugosus strain SPB7 16S ribosomal RNA gene, partial sequence	*B. rugosus*	2261	2261	97%	0.0	99.92%	1502	OR740574.1
B. paralicheniformis strain KJ-16 16S ribosomal RNA, partial sequence	*B. paralicheniformis*	2257	2257	100%	0.0	98.89%	1327	NR_137421.1
B. licheniformis strain ATCC 14580 chromosome, complete genome	*B. licheniformis*	2257	18028	100%	0.0	98.89%	4227253	CP140161.1
B.licheniformis gene for 16S rRNA	*B. licheniformis*	2257	2257	100%	0.0	98.89%	1552	X68416.1
B. licheniformis strain DSM 13 chromosome, complete genome	*B. licheniformis*	2257	18039	100%	0.0	98.89%	4243588	CP093290.1
B. licheniformis strain NCTC10341 genome assembly, chromosome: 1	*B. licheniformis*	2257	18028	100%	0.0	98.89%	4226784	LR134392.1
B. licheniformis DSM 13 = ATCC 14580, complete genome	*B. licheniformis* DSM 13 = ATCC 14580	2257	15776	100%	0.0	98.89%	4222645	AE017333.1
B. subtilis 16S ribosomal RNA gene, partial sequence	*B. subtilis*	2257	2257	97%	0.0	100.00%	1456	MW471618.1
B. licheniformis strain DSM 13 chromosome, complete genome	*B. licheniformis*	2257	18028	100%	0.0	98.89%	4229830	CP187612.1
B. licheniformis strain ATCC 14580 chromosome, complete genome	*B. licheniformis*	2257	18056	100%	0.0	98.89%	4239205	CP034569.1
B. licheniformis strain DSM 13 16S ribosomal RNA, partial sequence	*B. licheniformis*	2257	2257	100%	0.0	98.89%	1545	NR_118996.1
B. licheniformis ATCC 14580, complete genome	*B. licheniformis* DSM 13 = ATCC 14580	2257	15776	100%	0.0	98.89%	4222597	CP000002.3
B. subtilis strain DSM 10 16S ribosomal RNA gene, partial sequence	*B. subtilis*	2257	2257	97%	0.0	100.00%	1458	MK182759.1
B. velezensis strain NRRL B-41580 16S ribosomal RNA gene, partial sequence	*B. velezensis*	2255	2255	97%	0.0	99.76%	1508	KY694464.1
B. licheniformis strain ATCC 14580 16S ribosomal RNA, partial sequence	*B. licheniformis*	2252	2252	100%	0.0	98.81%	1545	NR_074923.1
B. glycinifermentans strain GO-13 16S ribosomal RNA gene, complete sequence	*B. glycinifermentans*	2252	2252	100%	0.0	98.81%	1554	KT005408.1
B. nakamurai strain NRRL B-41091 16S ribosomal RNA, partial sequence	*B. nakamurai*	2250	2250	97%	0.0	99.67%	1508	NR_151897.1
B. subtilis strain NBRC 13719 16S ribosomal RNA, partial sequence	*B. subtilis*	2248	2248	96%	0.0	100.00%	1475	NR_112629.1
B. spizizenii strain NBRC 101239 16S ribosomal RNA, partial sequence	*B. spizizenii*	2248	2248	96%	0.0	100.00%	1475	NR_112686.1
B. subtilis strain JCM 1465 16S ribosomal RNA, partial sequence	*B. subtilis*	2242	2242	96%	0.0	100.00%	1472	NR_113265.1
B. subtilis subsp. *spizizenii* strain BCRC 17366 16S ribosomal RNA gene, partial sequence	*B. spizizenii*	2239	2239	96%	0.0	100.00%	1468	EF433402.1
B. siamensis strain KCTC 13613 16S ribosomal RNA gene, partial sequence	*B. siamensis*	2235	2235	97%	0.0	99.59%	1492	KY643639.1
B. vallismortis strain NBRC 101236 16S ribosomal RNA, partial sequence	*B. vallismortis*	2233	2233	96%	0.0	99.75%	1475	NR_113994.1
B. amyloliquefaciens strain NBRC 15535 16S ribosomal RNA gene, partial sequence	*B. amyloliquefaciens*	2230	2230	97%	0.0	99.59%	1487	MK182997.1
B. siamensis KCTC 13613 16S ribosomal RNA gene, partial sequence	*B. siamensis* KCTC 13613	2226	2226	96%	0.0	99.75%	1473	MN176482.1

Supplementary Table S4. BLASTn results of the 16S rRNA gene sequence of BSS.246 against sequences from type material available in the NCBI database. The table presents the closest matches, including the scientific name, maximum and total alignment scores, query coverage, E-value, percentage identity, sequence length, and accession number. These results were used as the initial molecular evidence for the taxonomic assignment of BSS.246.

Description	Scientific Name	Max Score	Total Score	Query Cover	E value	Per. Ident	Acc. Len	Accession
B. ayatagriensis strain RMG6 16S ribosomal RNA gene, partial sequence	*B. ayatagriensis*	2837	2837	100%	0.0	99.81%	1545	PP494329.1
B. amyloliquefaciens DSM7 complete genome	*B. amyloliquefaciens* DSM 7 = ATCC 23350	2819	28185	100%	0.0	99.55%	3980199	FN597644.1
B. subtilis subsp. *subtilis* str. 168 genome assembly, chromosome: 1	*B. subtilis* subsp. *subtilis* str. 168	2813	27921	100%	0.0	99.48%	4215606	AL009126.3
B. subtilis subsp. *subtilis* strain KCTC 3135, complete genome	*B. subtilis* subsp. *subtilis*	2813	28008	100%	0.0	99.48%	4211343	CP015375.1
B. subtilis strain NCIB_3610 chromosome, complete genome	*B. subtilis*	2813	27989	100%	0.0	99.48%	4215634	CP130597.1
Chain a, 16S ribosomal RNA	*B. subtilis* subsp. *subtilis* str. 168	2813	2813	100%	0.0	99.48%	1554	6HA1_a
B. subtilis subsp. *subtilis* str. 168 chromosome, complete genome	*B. subtilis* subsp. *subtilis* str. 168	2813	27997	100%	0.0	99.48%	4227167	CP051860.2
Chain AA, 16S ribosomal RNA	*B. subtilis* subsp. *subtilis* str. 168	2813	2813	100%	0.0	99.48%	1555	3J9W_AA
B. subtilis subsp. *subtilis* strain DSM 10 chromosome, complete genome	*B. subtilis* subsp. *subtilis*	2813	27989	100%	0.0	99.48%	4215636	CP060710.1
B. subtilis strain NCIB 3610 chromosome, complete genome	*B. subtilis*	2813	27989	100%	0.0	99.48%	4215607	CP020102.1
B. subtilis strain DSM 10 chromosome, complete genome	*B. subtilis*	2813	27997	100%	0.0	99.48%	4215627	CP120681.2
B. subtilis subsp. *subtilis* str. 168, partial genome	*B. subtilis* subsp. *subtilis* str. 168	2813	27001	100%	0.0	99.48%	4215398	CP019662.1
B. subtilis subsp. *subtilis* strain 168 16S ribosomal RNA, complete sequence	*B. subtilis* subsp. *subtilis*	2813	2813	100%	0.0	99.48%	1550	NR_102783.2
B. subtilis subsp. *subtilis* str. 168, partial genome	*B. subtilis* subsp. *subtilis* str. 168	2813	27505	100%	0.0	99.48%	4215385	CP019663.1
B. subtilis subsp. *subtilis* str. 168 chromosome, complete genome	*B. subtilis* subsp. *subtilis* str. 168	2813	27997	100%	0.0	99.48%	4398844	CP052842.1
B. subtilis subsp. *subtilis* str. 168 chromosome, complete genome	*B. subtilis* subsp. *subtilis* str. 168	2813	27997	100%	0.0	99.48%	4215167	CP103783.1
B. subtilis subsp. *subtilis* str. 168 chromosome, complete genome	*B. subtilis* subsp. *subtilis* str. 168	2813	27997	100%	0.0	99.48%	4316079	CP053102.1
B. subtilis subsp. *subtilis* 6051-HGW, complete genome	*B. subtilis* subsp. *subtilis* 6051-HGW	2813	27921	100%	0.0	99.48%	4215610	CP003329.1
B. subtilis subsp. *subtilis* strain 168 chromosome, complete genome	*B. subtilis* subsp. *subtilis*	2813	27997	100%	0.0	99.48%	4215502	CP171645.1
B. subtilis subsp. *subtilis* str. 168, complete genome	*B. subtilis* subsp. *subtilis* str. 168	2813	27978	100%	0.0	99.48%	4215619	CP010052.1
B. subtilis subsp. *subtilis* NBRC 13719 DNA, complete genome	*B. subtilis* subsp. *subtilis*	2813	28002	100%	0.0	99.48%	4211090	AP019714.1
B. subtilis subsp. *subtilis* strain 168 16S ribosomal RNA gene, partial sequence	*B. subtilis* subsp. *subtilis*	2811	2811	100%	0.0	99.48%	1550	PQ164258.1

B. vallismortis strain DSM 11031 chromosome, complete genome	*B. vallismortis*	2808	27995	100%	0.0	99.42%	4286362	CP026362.1
Chain a, 16S rRNA	*B. subtilis* subsp. *subtilis* str. 168	2808	2808	100%	0.0	99.42%	1554	8BUU_a
B. subtilis strain NCIB 3610 chromosome, complete genome	*B. subtilis*	2808	28013	100%	0.0	99.42%	4210909	CP094361.1
B. vallismortis strain DV1-F-3 chromosome, complete genome	*B. vallismortis*	2808	27995	100%	0.0	99.42%	4234100	CP159908.1
B. subtilis subsp. *subtilis* str. 168 chromosome, complete genome	*B. subtilis* subsp. *subtilis* str. 168	2808	28002	100%	0.0	99.42%	4217427	CP147877.1
Chain A, 16S ribosomal RNA	*B. subtilis* subsp. *subtilis* str. 168	2806	2806	100%	0.0	99.48%	1544	5NJT_A
B. subtilis gene, 180 kilobase region of replication origin	*B. subtilis* subsp. *subtilis* str. 168	2806	11202	100%	0.0	99.35%	180136	D26185.1
B. mojavensis RO-H-1 = KCTC 3706 strain RO-H-1 chromosome, complete genome	*B. mojavensis* RO-H-1 = KCTC 3706	2802	25189	100%	0.0	99.35%	3976981	CP196936.1
B. subtilis subsp. *subtilis* str. 168 chromosome, complete genome	*B. subtilis* subsp. *subtilis* str. 168	2802	28008	100%	0.0	99.35%	4217417	CP136402.1
B. subtilis subsp. *subtilis* NCIB 3610 = ATCC 6051 = DSM 10 strain NCIB 3610 chromosome, complete genome	*B. subtilis* subsp. *subtilis* NCIB 3610 = ATCC 6051 = DSM 10	2802	28008	100%	0.0	99.35%	4211262	CP034484.1
B. inaquosorum strain KCTC 13429 chromosome, complete genome	*B. inaquosorum*	2802	25083	100%	0.0	99.35%	4350498	CP029465.1
B. subtilis strain DSM 10 chromosome, complete genome	*B. subtilis*	2802	28013	100%	0.0	99.35%	4211264	CP149576.1
Chain a, 16S ribosomal RNA	*B. subtilis* subsp. *subtilis* str. 168	2800	2800	100%	0.0	99.42%	1545	7QV2_a
B. cabrialesii strain TE3 16S ribosomal RNA, complete sequence	*B. cabrialesii*	2796	2796	100%	0.0	99.29%	1550	NR_180419.1
B. subtilis strain IAM 12118 16S ribosomal RNA, complete sequence	*B. subtilis*	2796	2796	100%	0.0	99.29%	1550	NR_112116.2
B. subtilis strain IAM 12118 16S ribosomal RNA gene, partial sequence	*B. subtilis*	2796	2796	100%	0.0	99.29%	1550	MZ315033.1
B. cabrialesii strain TE3 chromosome, complete genome	*B. cabrialesii*	2796	27930	100%	0.0	99.29%	4125766	CP096889.1
B. cabrialesii strain TE3 16S ribosomal RNA gene, partial sequence	*B. cabrialesii*	2796	2796	100%	0.0	99.29%	1550	MK462260.1
B. subtilis gene for 16S ribosomal RNA, partial sequence	*B. subtilis*	2796	2796	100%	0.0	99.29%	1553	AB042061.1
B. velezensis strain BCRC 17467 16S ribosomal RNA gene, partial sequence	*B. velezensis*	2793	2793	99%	0.0	99.54%	1535	EF433407.1
B. subtilis subsp. *spizizenii* TU-B-10, complete genome	*B. spizizenii* TU-B-10	2791	27880	100%	0.0	99.22%	4207222	CP002905.1
B. rugosus strain SPB7 16S ribosomal RNA gene, partial sequence	*B. rugosus*	2791	2791	100%	0.0	99.22%	1548	MT554518.1
B. rugosus strain SPB7 16S ribosomal RNA, partial sequence	*B. rugosus*	2791	2791	100%	0.0	99.22%	1548	NR_181236.1
Chain a, 16S rRNA	*B. subtilis* subsp. *subtilis* str. 168	2785	2785	99%	0.0	99.48%	1533	6HTQ_a
B. subtilis DNA for phoB-rrnE-groESL region, complete cds	*B. subtilis*	2785	2785	100%	0.0	99.16%	36308	D88802.1
B. subtilis genome, trnI-feuABC region	*B. subtilis* subsp. *subtilis* str. 168	2785	5553	100%	0.0	99.16%	15467	D84213.1

B. spizizenii ATCC 6633 = JCM 2499 strain ATCC 6633 chromosome, complete genome	*B. spizizenii* ATCC 6633 = JCM 2499	2785	27858	100%	0.0	99.16%	4045538	CP034943.1
B. halotolerans strain DSM 8802 16S ribosomal RNA, partial sequence	*B. halotolerans*	2784	2784	100%	0.0	99.29%	1545	NR_115063.1
B. inaquosorum strain BGSC 3A28 16S ribosomal RNA, partial sequence	*B. inaquosorum*	2780	2780	99%	0.0	99.28%	1538	NR_104873.1
B. stercoris strain JCM 30051 16S ribosomal RNA gene, partial sequence	*B. stercoris*	2778	2778	100%	0.0	99.22%	1548	PP239385.1
B. subtilis genes for ribosomal proteins L13 and S9, putative cell wall hydrolase CwlD, gerD protein, 16S ribosomal RNA and 23S ribosomal RNA	*B. subtilis* subsp. *subtilis* str. 168	2778	2778	100%	0.0	99.10%	13119	D64126.1
B. vallismortis strain DSM 11031 16S ribosomal RNA, partial sequence	*B. vallismortis*	2776	2776	99%	0.0	99.41%	1530	NR_024696.1
B. subtilis 25 kb genomic DNA segment (from sspE to katA)	*B. subtilis* subsp. *subtilis* str. 168	2767	2767	100%	0.0	98.97%	24524	Z82044.1
B. mojavensis strain IFO 15718 16S ribosomal RNA, partial sequence	*B. mojavensis*	2758	2758	99%	0.0	99.28%	1526	NR_024693.1
B. velezensis strain NRRL B-41580 16S ribosomal RNA gene, partial sequence	*B. velezensis*	2752	2752	97%	0.0	99.60%	1508	KY694464.1
B. subtilis strain DSM 10 16S ribosomal RNA, partial sequence	*B. subtilis*	2747	2747	98%	0.0	99.34%	1517	NR_027552.1
B. nakamurai strain NRRL B-41091 16S ribosomal RNA, partial sequence	*B. nakamurai*	2741	2741	97%	0.0	99.47%	1508	NR_151897.1
B. subtilis strain DSM 10 16S ribosomal RNA gene, partial sequence	*B. subtilis*	2739	2739	98%	0.0	99.34%	1513	MW049219.1
B. atrophaeus strain JCM 9070 16S ribosomal RNA, partial sequence	*B. atrophaeus*	2737	2737	98%	0.0	99.27%	1515	NR_024689.1
B. siamensis strain KCTC 13613 16S ribosomal RNA gene, partial sequence	*B. siamensis*	2736	2736	96%	0.0	99.80%	1492	KY643639.1
B. nematocida strain B-16 16S ribosomal RNA, partial sequence	*B. nematocida*	2732	2732	98%	0.0	99.27%	1511	NR_115325.1
B. subtilis strain DSM 10 16S ribosomal RNA gene, partial sequence	*B. subtilis*	2732	2732	98%	0.0	99.34%	1510	MN077147.1
B. siamensis KCTC 13613 strain PD-A10 16S ribosomal RNA, partial sequence	*B. siamensis* KCTC 13613	2730	2730	98%	0.0	99.14%	1525	NR_117274.1
B. amyloliquefaciens strain NBRC 15535 16S ribosomal RNA gene, partial sequence	*B. amyloliquefaciens*	2728	2728	96%	0.0	99.80%	1487	MK182997.1
B. stercoris strain JCM 30051 16S ribosomal RNA, partial sequence	*B. stercoris*	2724	2724	97%	0.0	99.27%	1508	NR_180796.1
B. tequilensis strain KCTC 13622(T) 16S ribosomal RNA gene, partial sequence	*B. tequilensis*	2724	2724	98%	0.0	99.14%	1518	MW009674.1
B. subtilis subsp. *stercoris* strain JCM 30051 16S ribosomal RNA gene, partial sequence	*B. stercoris*	2724	2724	97%	0.0	99.27%	1508	MN536904.1
B. subtilis strain IAM 12118 16S ribosomal RNA gene, partial sequence	*B. subtilis*	2723	2723	97%	0.0	99.34%	1519	MK267098.1
B. subtilis strain IAM 12118 16S ribosomal RNA gene, partial sequence	*B. subtilis*	2723	2723	98%	0.0	99.14%	1521	PV523860.1
B. subtilis partial 16S rRNA gene, type strain DSM 10T	*B. subtilis*	2719	2719	97%	0.0	99.33%	1502	LN681568.1

B. subtilis subsp. *spizizenii* strain NRRL B-23049 16S ribosomal RNA gene, partial sequence	*B. spizizenii*	2719	2719	97%	0.0	99.20%	1508	MN536905.1
B. subtilis strain IAM 12118 16S ribosomal RNA gene, partial sequence	*B. subtilis*	2715	2715	98%	0.0	99.07%	1517	PV616805.1
B. subtilis gene for 16S rRNA, partial sequence, strain: ATCC 6633	*B. spizizenii* ATCC 6633 = JCM 2499	2712	2712	97%	0.0	99.14%	1507	AB018486.1
B. subtilis subsp. *subtilis* str. 168 16S ribosomal RNA gene, partial sequence	*B. subtilis* subsp. *subtilis* str. 168	2712	2712	98%	0.0	99.07%	1518	PV257656.1
B. inaquosorum strain BGSC 3A28 16S ribosomal RNA gene, partial sequence	*B. inaquosorum*	2706	2706	97%	0.0	99.20%	1501	PX218924.1
B. glycinifermentans strain GO-13 16S ribosomal RNA gene, complete sequence	*B. glycinifermentans*	2699	2699	100%	0.0	98.19%	1554	KT005408.1
B. rugosus strain SPB7 16S ribosomal RNA gene, partial sequence	*B. rugosus*	2695	2695	97%	0.0	99.07%	1502	OR740574.1
B. siamensis KCTC 13613 16S ribosomal RNA gene, partial sequence	*B. siamensis* KCTC 13613	2693	2693	95%	0.0	99.66%	1473	MN176482.1
B. amyloliquefaciens strain NBRC 15535 16S ribosomal RNA, partial sequence	*B. amyloliquefaciens*	2687	2687	95%	0.0	99.53%	1475	NR_112685.1
B. amyloliquefaciens strain NBRC 15535 16S ribosomal RNA, partial sequence	*B. amyloliquefaciens*	2686	2686	95%	0.0	99.59%	1472	NR_041455.1
B. siamensis KCTC 13613 16S ribosomal RNA gene, partial sequence	*B. siamensis* KCTC 13613	2684	2684	95%	0.0	99.59%	1474	PQ764821.1
B. licheniformis strain ATCC 14580 chromosome, complete genome	*B. licheniformis*	2682	21382	100%	0.0	98.00%	4227253	CP140161.1
B. licheniformis strain DSM 13 chromosome, complete genome	*B. licheniformis*	2682	21393	100%	0.0	97.99%	4243588	CP093290.1
B. licheniformis strain NCTC10341 genome assembly, chromosome: 1	*B. licheniformis*	2682	21382	100%	0.0	98.00%	4226784	LR134392.1
B. licheniformis DSM 13 = ATCC 14580, complete genome	*B. licheniformis* DSM 13 = ATCC 14580	2682	18721	100%	0.0	98.00%	4222645	AE017333.1
B. licheniformis strain DSM 13 chromosome, complete genome	*B. licheniformis*	2682	21376	100%	0.0	98.00%	4229830	CP187612.1
B. licheniformis strain ATCC 14580 chromosome, complete genome	*B. licheniformis*	2682	21387	100%	0.0	97.99%	4239205	CP034569.1
B. licheniformis ATCC 14580, complete genome	*B. licheniformis* DSM 13 = ATCC 14580	2682	18721	100%	0.0	98.00%	4222597	CP000002.3
B. licheniformis gene for 16S rRNA	*B. licheniformis*	2673	2673	100%	0.0	97.93%	1552	X68416.1
B. amyloliquefaciens strain BCRC 11601 16S ribosomal RNA, partial sequence	*B. amyloliquefaciens*	2673	2673	95%	0.0	99.52%	1468	NR_116022.1
B. licheniformis strain DSM 13 16S ribosomal RNA, partial sequence	*B. licheniformis*	2673	2673	100%	0.0	97.93%	1545	NR_118996.1
B. vallismortis strain NBRC 101236 16S ribosomal RNA, partial sequence	*B. vallismortis*	2671	2671	95%	0.0	99.32%	1475	NR_113994.1
B. subtilis strain NBRC 13719 16S ribosomal RNA, partial sequence	*B. subtilis*	2669	2669	95%	0.0	99.32%	1475	NR_112629.1
B. velezensis strain CR-502 16S ribosomal RNA gene, partial sequence	*B. velezensis*	2669	2669	95%	0.0	99.39%	1472	MG846018.1

B. atrophaeus strain NBRC 15539 16S ribosomal RNA, partial sequence	*B. atrophaeus*	2665	2665	95%	0.0	99.25%	1475	NR_112723.1
B. subtilis strain JCM 1465 16S ribosomal RNA, partial sequence	*B. subtilis*	2663	2663	95%	0.0	99.32%	1472	NR_113265.1
B. mojavensis strain NBRC 15718 16S ribosomal RNA, partial sequence	*B. mojavensis*	2663	2663	95%	0.0	99.25%	1475	NR_112725.1
B. licheniformis strain ATCC 14580 16S ribosomal RNA, partial sequence	*B. licheniformis*	2662	2662	100%	0.0	97.80%	1545	NR_074923.1

Supplementary Table S5. Digital DNA–DNA hybridization (dDDH) results for BSS.245 and the selected reference genomes. The table presents the query and subject strains, dDDH estimates calculated using the generalized distance formulas (d_0, d_4, and d_6), corresponding confidence intervals (C.I.), and differences in G+C content. The analyses were performed using the Type Strain Genome Server (TYGS) and were used to assess the genomic relatedness of BSS.245 to closely related *Bacillus* strains and to support its species-level taxonomic assignment. A dDDH value of ≥70% was considered indicative of assignment to the same species.

**Query strain**	**Type strain**	**dDDH (d_0, in %)**	**C.I. (d_0, in %)**	**dDDH (d_4, in %)**	**C.I. (*d4*, in %)**	**dDDH (d_6, in %)**	**C.I. (d_6, in %)**	**G+C content difference (in %)**
**BSS.245**	*B. subtilis* subsp. *subtilis* 168	91.9	[89.0 - 94.1]	89.8	[87.4 - 91.7]	93.9	[91.8 - 95.6]	0.16
**BSS.245**	*B. stercoris* Mal05	87.7	[84.2 - 90.5]	62.6	[59.7 - 65.4]	85.7	[82.6 - 88.4]	0.01
**BSS.245**	*B. spizizenii* TU-B-10	86.7	[83.0 - 89.6]	50.5	[47.8 - 53.1]	81.3	[77.9 - 84.2]	0.15
**BSS.245**	*B. inaquosorum* A65.1	83.4	[79.5 - 86.6]	50.3	[47.6 - 52.9]	78.5	[75.0 - 81.5]	0.36
**BSS.245**	*B. rugosus* A78.1	88	[84.5 - 90.8]	48.8	[46.2 - 51.4]	81.8	[78.4 - 84.7]	0.19
**BSS.245**	*B. cabrialesii* TE3	87.5	[84.0 - 90.4]	47.7	[45.1 - 50.3]	81	[77.6 - 84.0]	0.54
**BSS.245**	*B. tequilensis* EA-CB0015	78.2	[74.2 - 81.7]	45	[42.5 - 47.6]	72.2	[68.7 - 75.4]	0.06
**BSS.245**	*B. vallismortis* Bac111	77.8	[73.8 - 81.3]	42.8	[40.3 - 45.3]	70.9	[67.5 - 74.2]	0.48
**BSS.245**	*B. mexicanus* MoR6	56.7	[53.1 - 60.2]	39.8	[37.4 - 42.4]	53.1	[50.0 - 56.2]	2.61
**BSS.245**	*B. halotolerans* ZB201702	84.4	[80.7 - 87.6]	32.7	[30.3 - 35.2]	70.4	[66.9 - 73.6]	0.13
**BSS.245**	*B. mojavensis* RO-H-1	81.2	[77.3 - 84.6]	32.5	[30.1 - 35.0]	67.8	[64.4 - 71.0]	0.09
**BSS.245**	*B. atrophaeus* SW	46.9	[43.5 - 50.4]	22.1	[19.8 - 24.5]	38.2	[35.2 - 41.2]	0.51
**BSS.245**	*B. siamensis* M54	31.9	[28.5 - 35.5]	20.6	[18.4 - 23.0]	27.9	[25.0 - 31.0]	2.86
**BSS.245**	*B. amyloliquefaciens* GKT04	32.5	[29.1 - 36.0]	20.6	[18.4 - 23.0]	28.3	[25.4 - 31.4]	2.61
**BSS.245**	*B. velezensis* FZB42	33.2	[29.8 - 36.8]	20.6	[18.4 - 23.0]	28.8	[25.9 - 31.9]	2.8
**BSS.245**	*B. nakamurai* NRRL B-41091	33.8	[30.4 - 37.4]	20.4	[18.1 - 22.8]	29.1	[26.2 - 32.2]	1.58
**BSS.245**	*B. ayatagriensis* RMG6	32.7	[29.4 - 36.3]	20.3	[18.1 - 22.8]	28.4	[25.5 - 31.5]	2.96
**BSS.245**	*B. paralicheniformis* Bac84	16.8	[13.8 - 20.3]	18.9	[16.7 - 21.3]	16.5	[14.0 - 19.5]	2.17
**BSS.245**	*B. glycinifermentans* SRCM103574	16	[13.1 - 19.5]	18.9	[16.7 - 21.3]	15.9	[13.4 - 18.8]	2.3
**BSS.245**	*B. licheniformis* ATCC 14580	17	[13.9 - 20.5]	18.4	[16.3 - 20.8]	16.6	[14.1 - 19.6]	2.53

Supplementary Table S6. Digital DNA–DNA hybridization (dDDH) results for BSS.246 and the selected reference genomes. The table presents the query and subject strains, dDDH estimates calculated using the generalized distance formulas (d_0, d_4, and d_6), corresponding confidence intervals (C.I.), and differences in G+C content. The analyses were performed using the Type Strain Genome Server (TYGS) and were used to assess the genomic relatedness of BSS.246 to closely related *Bacillus* strains and to support its species-level taxonomic assignment. A dDDH value of ≥70% was considered indicative of assignment to the same species.

Query strain	Type strain	dDDH (d_0, in %)	C.I. (d_0, in %)	dDDH (d_4, in %)	C.I. (*d4*, in %)	dDDH (d_6, in %)	C.I. (d_6, in %)	G+C content difference (in %)
**BSS.246**	*B. ayatagriensis* RMG6	96.1	[94.1 - 97.4]	95.5	[94.0 - 96.6]	97.5	[96.2 - 98.3]	0.05
**BSS.246**	*B. velezensis* FZB42	92.8	[90.0 - 94.9]	80	[77.1 - 82.7]	93.3	[91.0 - 95.0]	0.11
**BSS.246**	*B. amyloliquefaciens* GKT04	90.3	[87.1 - 92.7]	79.6	[76.6 - 82.2]	91.2	[88.6 - 93.3]	0.3
**BSS.246**	*B. siamensis* M54	89.6	[86.3 - 92.1]	56.8	[54.1 - 59.6]	85.8	[82.6 - 88.4]	0.05
**BSS.246**	*B. nakamurai* NRRL B-41091	71.4	[67.4 - 75.0]	30.9	[28.5 - 33.4]	59.7	[56.4 - 62.8]	1.33
**BSS.246**	*B. inaquosorum* A65.1	33.9	[30.5 - 37.4]	21.1	[18.8 - 23.5]	29.4	[26.5 - 32.5]	2.56
**BSS.246**	*B. tequilensis* EA-CB0015	33	[29.6 - 36.6]	21.1	[18.8 - 23.5]	28.8	[25.9 - 31.9]	2.85
**BSS.246**	*B. atrophaeus* SW	30.4	[27.0 - 34.0]	21	[18.8 - 23.4]	27	[24.1 - 30.1]	3.42
**BSS.246**	*B. rugosus* A78.1	31.7	[28.4 - 35.3]	20.9	[18.6 - 23.3]	27.9	[25.0 - 31.0]	2.72
**BSS.246**	*B. stercoris* Mal05	32.7	[29.4 - 36.3]	20.8	[18.6 - 23.2]	28.5	[25.6 - 31.6]	2.93
**BSS.246**	*B. cabrialesii* TE3	33.5	[30.2 - 37.1]	20.8	[18.6 - 23.2]	29.1	[26.1 - 32.2]	2.37
**BSS.246**	*B. spizizenii* TU-B-10	32.9	[29.5 - 36.4]	20.8	[18.6 - 23.2]	28.6	[25.7 - 31.7]	2.76
**BSS.246**	*B. mojavensis* RO-H-1	32.8	[29.5 - 36.4]	20.7	[18.4 - 23.1]	28.6	[25.7 - 31.7]	2.82
**BSS.246**	*B. halotolerans* ZB201702	33.5	[30.1 - 37.1]	20.7	[18.4 - 23.1]	29	[26.1 - 32.1]	2.78
**BSS.246**	*B. subtilis* subsp. *subtilis* 168	32.8	[29.4 - 36.4]	20.7	[18.4 - 23.1]	28.5	[25.6 - 31.6]	3.07
**BSS.246**	*B. vallismortis* Bac111	32.1	[28.7 - 35.7]	20.6	[18.4 - 23.0]	28	[25.1 - 31.2]	2.43
**BSS.246**	*B. licheniformis* ATCC 14580	16	[13.0 - 19.4]	19.6	[17.4 - 22.0]	15.9	[13.4 - 18.8]	0.38
**BSS.246**	*B. glycinifermentans* SRCM103574	16.3	[13.3 - 19.8]	19.2	[17.1 - 21.6]	16.2	[13.6 - 19.1]	0.61

Supplementary Table S7. Prediction of genes and metabolic traits associated with biological control, plant growth promotion, plant signaling and systemic resistance induction, rhizospheric competence, and stress response in *Bacillus subtilis* BSS.245 and *Bacillus ayatagriensis* BSS.246. The presence (+) or absence (−) of genes or pathways related to the biosynthesis of secondary metabolites, hydrolytic enzymes, volatile organic compounds (VOCs), nutrient acquisition, phytohormone modulation, root colonization, substrate utilization, antioxidant defense, osmoregulation, and dormancy/sporulation is indicated.

**Genes function**	**Category**	**Compound / mechanism**	**BSS.245**	**BSS.246**
**Biological control**	Secondary metabolites	Bacillaene	+	+
		Bacillibactin	+	+
		Bacilysin	+	+
		Butirosin	-	+
		Difficidin	-	+
		Fengycin	+	+
		Locillomycin	-	+
		Macrolactin H	-	+
		Plipastatin	+	-
		Subtilomycin	+	-
		Subtilosin A	+	-
		Surfactin	+	+
	Hydrolytic enzymes	β-Glucanase	+	+
		Chitinase	-	+
		Chitosanase	+	+
	Indirect mechanisms	Siderophores	+	+

	VOCs	2,3-Butanediol	+	+
		Acetoin	+	+
**Plant growth promotion**	Nutrient acquisition	Acetic acid	+	+
		Aconitic acid	+	+
		Butyric acid	+	+
		Carbonic acid	+	+
		Citric acid	+	+
		Formic acid	-	+
		Fumaric acid	+	+
		Gluconic acid	+	+
		Isocitric acid	+	+
		Ketoglucaric acid	+	+
		Lactic acid	+	+
		Malic acid	+	+
		Malonic acid	+	+
		Propionic acid	+	+
		Succinic acid	+	+
		Tartaric acid	+	+
		Valeric acid	+	+
		Alkanesulfonate degradation	+	+

		Sulfate reduction	+	+
	Phytohormone modulation	Tryptophan biosynthesis	+	+
		Auxin production	+	+
		Cytokinin production	+	+
		Spermidine production	+	+
		GABA metabolism	+	+
**Plant signaling and systemic resistance induction**	Root colonization	EPS biosynthesis	+	+
	VOCs	Isoprene production	+	+
**Rhizospheric competence**	Acid assimilation	3-Oxoadipate	+	+
	Amino acid and relative compounds assimilation	Gamma-aminobutyric acid (GABA)	+	-
		Glutamine	+	+
		Proline	+	+
	Carbohydrate utilization	Inositol	+	+
		Fructose	+	-
		Maltose	+	-
		Mannitol	+	+
		Sucrose	+	+
		Trehalose	+	+
		Xylan	+	+
**Stress response**	Antioxidant enzymes	Alkyl hydroperoxide reductase	+	+

		Catalase	+	+
		Glutathione peroxidase	+	+
		Methionine sulfoxide reductase	+	+
		Superoxide dismutase	+	+
		Thioredoxin reductase	+	+
	Osmoregulation	Glycine betaine	+	+
		Proline	+	+
		Trehalose	+	+
	Dormancy and sporulation	Endospore formation	+	+

Supplementary Text S1

Literature search supporting the first report of antifungal activity of *Bacillus ayatagriensis*

To assess whether antifungal activity or biocontrol potential had previously been reported for *Bacillus ayatagriensis*, a literature search was conducted using Scholar Google, PubMed, Scopus, Web of Science, and ScienceDirect databases. The search was performed on August 31, 2026, and included records published up to this date.

The search strategy was designed to identify studies reporting antifungal activity, antagonistic interactions with fungi, biological control of fungal phytopathogens, or related functional activities specifically associated with *B. ayatagriensis*. The core search strategy combined the species name with terms related to antifungal and biocontrol activity. The following terms were used in different combinations: "*Bacillus ayatagriensis*" AND ("antifungal activity" OR "antagonism" OR "biocontrol" OR "biological control" OR "plant pathogen" OR "phytopathogen").

Where supported by the respective database interfaces, searches were performed in title, abstract, and keyword fields. Google Scholar was searched using the corresponding full-text query combinations because of its different search interface and indexing structure. The search was not restricted by geographic region, host plant, fungal genus, or publication type at the initial identification stage.

Records were imported into a single screening list, and duplicate records were identified based on title, authors, DOI, and/or bibliographic information and removed before screening. Titles and abstracts were subsequently screened for relevance. Full-text articles were assessed when the title and abstract did not provide sufficient information to determine eligibility.

Studies were considered eligible when they: (i) specifically investigated *B. ayatagriensis*; and (ii) reported experimental evaluation of antifungal activity, antagonistic activity against fungi, inhibition of phytopathogenic fungi, or biocontrol activity involving fungal pathogens. Studies were excluded when they: (i) did not investigate *B. ayatagriensis*; (ii) did not provide experimental evidence of antifungal, antagonistic, or fungal biocontrol activity; (iii) addressed only genomic, taxonomic, ecological, or biochemical characterization without evaluating antifungal or biocontrol activity; or (iv) were published before the formal description of *B. ayatagriensis* and therefore did not provide evidence specifically attributable to the formally recognized species.

Importantly, studies reporting antibacterial activity, inhibition of human pathogens, plant growth-promoting traits, enzyme production, or other functional properties were not considered evidence of antifungal activity against phytopathogenic fungi unless an experimental fungal assay was explicitly performed. This distinction was applied because the novelty claim in the present study refers specifically to the experimental antifungal activity of *B. ayatagriensis* against phytopathogenic fungi.

A total of 26 records were initially identified across the databases. After removal of 19 duplicate records, seven unique records remained for title and abstract screening. Six records were excluded during screening because they did not investigate *B. ayatagriensis* (n = 5) or were published before the formal description of *B. ayatagriensis* (n = 1). One record was retained for full-text relevance assessment. These studies are described in Supplementary Table S8.

Supplementary Table S8. Records identified and screened during the literature search conducted to assess the novelty of the antifungal activity of *Bacillus ayatagriensis*. These studies were excluded during title and abstract screening

No.	Reference	Title	Reason for exclusion
**1**	Ceccarini and Cohen (1967)	Germination inhibitor from the cellular slime mould *Dictyostelium discoideum*	The study was published before the formal description of *B. ayatagriensis*
**2**	Jurry et al. (2026)	Morpho-biochemical and molecular identification of *Bacillus licheniformis* and *Bacillus cereus* isolates from sorghum (*Sorghum bicolor* L.) rhizosphere	The study did not investigate *B. ayatagriensis*.
**3**	Chernyshova et al. (2025)	Characterization of *Bacillus velezensis* EV17 and K-3618 and their polyketide antibiotic oxydifficidin, an inhibitor of prokaryotic translation with low cytotoxicity	The study did not investigate *B. ayatagriensis*.
**4**	Hassan-Dalléac et al. (2026)	Soil microbes are the tiny bioengineers running Earth's underground factory	The study did not investigate *B. ayatagriensis*.
**5**	Potshangbam et al. (2026)	Characterization of *Bacillus siamensis* sdc15 from *Oryza sativa* L.(Black Rice) seed reveals multifunctional plant growth-promoting and biocontrol traits	The study did not investigate *B. ayatagriensis*.
**6**	Xu et al. (2025)	Pea–cucumber crop rotation suppresses *Fusarium* pathogens by reshaping soil microbial communities and enhancing nutrient availability	The study did not investigate *B. ayatagriensis*.

The final assessment of the retained study indicated that the previously reported activities of *B. ayatagriensis* were limited to plant growth-promoting activity and inhibition of human-associated pathogens, with no experimental evaluation of its activity against phytopathogenic fungi (Das et al 2025). Therefore, to the best of our knowledge and based on the search strategy and databases described above, no previous study identified in this search has experimentally evaluated the antifungal activity of *B. ayatagriensis* against phytopathogenic fungi.

The present literature search was performed specifically to support the novelty assessment of the antifungal activity reported in this study and was not intended to constitute a systematic review or meta-analysis. The complete identification, screening, exclusion, and inclusion workflow is presented in Supplementary Figure S1.

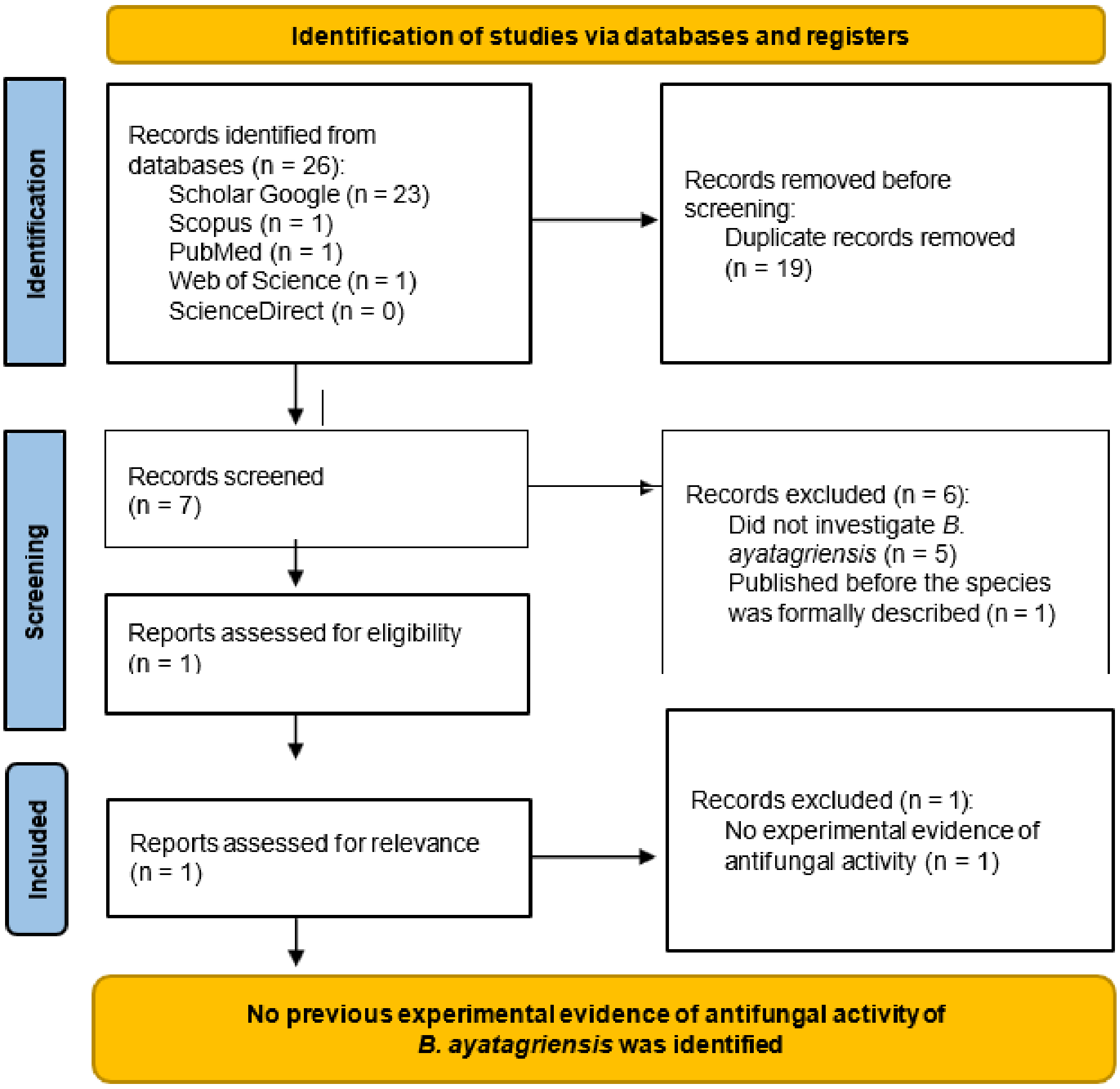

Supplement: Supplementary file 1 [file 10142_2026_2063_MOESM1_ESM.pdf]
